# AND‐Logic‐Gated Aptamer Switch for Precise Targeting and Regulation of RNA G‐Quadruplexes

**DOI:** 10.1002/anie.4594145

**Published:** 2026-05-13

**Authors:** Dan Wang, Ying Feng, Chun Kit Kwok

**Affiliations:** ^1^ Department of Chemistry and State Key Laboratory of Marine Environmental Health City University of Hong Kong Hong Kong China; ^2^ Shenzhen Research Institute of City University of Hong Kong Shenzhen China

**Keywords:** AND logic gate, gene regulation, L‐RNA aptamers, nucleic acids, RNA G‐quadruplexes

## Abstract

RNA G‐quadruplexes (rG4s) play critical roles in gene regulation and cancer progression, yet their precise manipulation in tumor cells remains challenging. rG4‐targeting L‐RNA aptamers are an emerging class of ligands with exceptional affinity for rG4s; however, their lack of cell type‐specific delivery hinders their regulatory and therapeutic potential. Herein, we engineer an activatable bispecific aptamer switch, termed the Allosteric RNA G‐quadruplex ON‐switch (ARGON), which integrates an rG4‐targeting L‐RNA Apt.4‐1c module (masked by a glutathione (GSH)‐cleavable lock strand) with a tumor receptor‐targeting Sgc8 DNA aptamer to precisely target rG4s and regulate downstream cellular activities within tumor cells. The AND logic‐gated ARGON is activated exclusively in tumor cells that exhibit both tumor receptor overexpression and elevated GSH levels. Following cellular uptake, GSH‐triggered lock cleavage exposes L‐Apt.4‐1c's rG4‐binding domain, enabling binding oncogenic *Bcl2* rG4. Then activated ARGON regulates rG4‐associated tumor cellular functions while sparing normal cells. Besides, we apply ARGON to target human telomerase RNA component (*hTERC*) rG4 to show our method's generality. Collectively, by integrating cell‐surface addressing with intracellular environmental sensing, our work reports an “old‐chemistry‐new‐trick” framework for regulating nucleic acid structures, enabling conditional targeting of cellular RNA structures with minimal off‐target effects and propelling aptamer‐based precision biomedicine forward.

## Graphical abstract

An AND‐logic‐gated aptamer switch enables tumor‐selective activation of RNA G‐quadruplex (rG4)‐targeting function. By combining cell‐surface recognition and intracellular redox sensing, the system achieves precise regulation of disease‐relevant RNA structures exclusively in tumor cells, with minimal off‐target effects in normal cells, offering a scalable molecular tool for future conditional nucleic acid regulation.

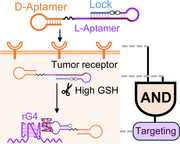

## 1 Introduction

RNA G‐quadruplexes (rG4s) are non‐canonical nucleic acid secondary structures formed by guanine‐rich segments via Hoogsteen hydrogen bonding [1, 2, 3]. rG4s are predominantly localized in untranslated regions, non‐coding RNAs, and coding sequences of mRNAs [4, 5]. Cellular rG4s exhibit dynamic behavior and play versatile roles in RNA turnover, phase separation, RNA splicing, and protein translation, rendering them attractive therapeutic targets [6, 7, 8, 9, 10, 11]. Hence, targeting and modulating rG4s with specific ligands can influence gene expression [12], protein interactions [13], cellular stress response [14, 15], and the onset and progression of various diseases.

G4‐targeting L‐RNA aptamers, selected and developed by our group, are a novel class of ligands for specific and efficient targeting of D‐rG4s via non‐Watson−Crick base pairing and tertiary structural complementarity [16, 17, 18]. Unlike CRISPR or antisense oligonucleotides (ASOs), which target specific sequences related to rG4s [19, 20], L‐RNA aptamer targets the rG4 tertiary structure, enabling regulation of genes where structural formation acts as the disease‐relevant switch, a layer of control inaccessible to sequence‐only binders. Furthermore, while CRISPR involves permanent genomic editing and ASOs often trigger irreversible transcript degradation, our L‐RNA aptamer modulates translation reversibly via steric hindrance, offering a safer, tunable intervention for transient therapeutic needs without off‐target genomic effects. Finally, the inherent nuclease resistance of L‐RNA ensures intracellular stability without the possible chemical modifications required for guide RNA or DNA ASOs, thereby simplifying the design while maintaining efficacy. In comparison to traditional rG4 ligands such as small molecules and antibodies [21], L‐RNA aptamers exhibit nuclease resistance and selective discrimination between rG4 subtypes [22, 23, 24]. And crucially, they perform high modifiability, programmability, and tunability for plug‐and‐play engineering [25, 26, 27], as exemplified in nucleic acid nanotechnology [28, 29, 30, 31].

However, L‐RNA aptamers are negatively charged biomacromolecules that cannot efficiently enter cells autonomously. Although transfection using Lipofectamine or cell‐penetrating peptides has been reported to assist L‐RNA aptamer access to intracellular rG4s [32], these methods lack the ability to automatically deliver L‐RNA aptamers to specific cell types. This limitation hinders the advancement of rG4 targeting and modulation research in complex multicellular contexts. For instance, while a specific oncogenic rG4 in tumor cells requires targeting and modulation, the same rG4 in normal cells should remain unaffected. However, such “on‐demand” delivery or activation strategies for the specific targeting of rG4s in user‐defined cells remain to be explored.

**SCHEME 1:**
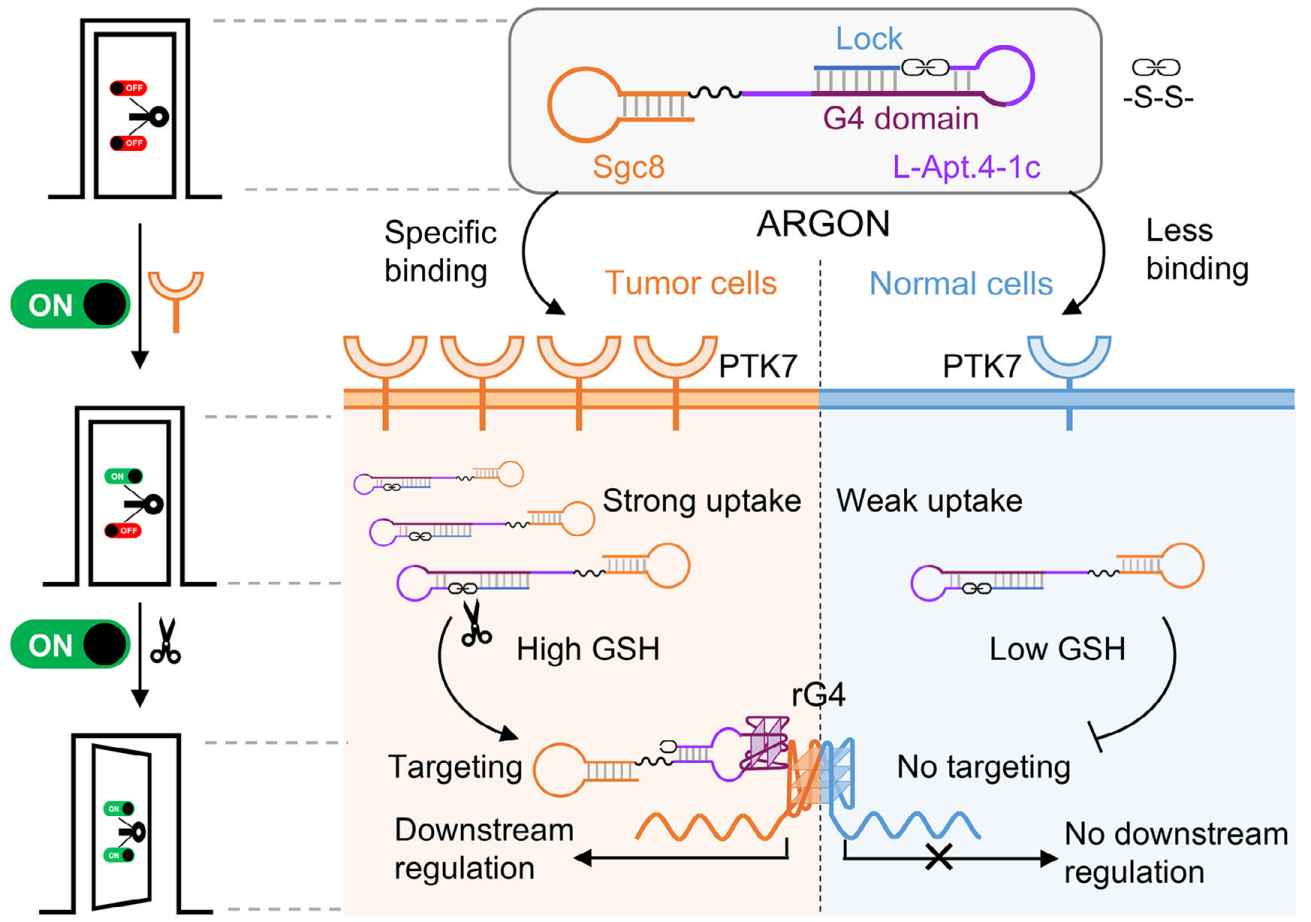
Design principles and workflow of transmembrane‐targeting ARGON (Allosteric RNA G‐quadruplex ON‐switch). ARGON is equipped with two switch buttons (Sgc8‐PTK7 recognition and GSH‐lock interaction) to control the access to targeting tumor cellular rG4s, including an oncogenic *Bcl2* rG4 of interest and scalable *hTERC* rG4, and then regulating downstream molecular interactions and biological activities.

D‐type aptamers are single‐stranded DNA or RNA oligonucleotides that are able to bind targets with high affinity and specificity [33, 34, 35, 36, 37]. They have been broadly utilized in biomarker discovery, bioimaging, molecular diagnostics, and targeted therapy, given their proven utility as potent tools for active cellular recognition and targeting over the past decade [36, 38, 39, 40, 41, 42, 43]. To address the issue mentioned previously, we have designed the first heterodimeric aptamer conjugate comprising a D‐type DNA aptamer (targeting a cell‐surface protein) and an activatable L‐type RNA aptamer (targeting intracellular rG4s), enabling both cell‐specific delivery and allosteric activation within the tumor microenvironment (TME)—a milieu characterized by elevated intracellular glutathione (GSH) levels. As proof of concept, a heterodimeric aptamer is engineered using the previously reported L‐Apt.4‐1c and Sgc8 aptamer [22, 44], termed Allosteric RNA G‐quadruplex ON‐switch (ARGON), to target an oncogenic *Bcl2* rG4 of interest and a scalable human telomerase RNA component (*hTERC*) rG4, in protein tyrosine kinase 7 (PTK7)‐overexpressed tumor cells (Scheme 1). Both aptamers have been originally identified through Systematic Evolution of Ligands by EXponential enrichment (SELEX) coupled with Next‐Generation Sequencing (NGS) [17, 22, 44, 45]. The two rG4s have been identified high‐affinity binding targets before [22]. The construction of ARGON involves a two‐step strategy: first, a lock strand bearing a terminal disulfide group is hybridized to the active rG4‐binding domain of L‐Apt.4‐1c—which coincides with its own G4‐forming region—thereby masking its functionality; second, this locked L‐RNA complex is covalently conjugated to the Sgc8 aptamer via click chemistry. Inspired by previously reported “dual‐key–one‐lock” mechanisms [46, 47, 48, 49, 50], we propose that the Sgc8‐PTK7 interaction and GSH‐mediated lock cleavage can act as two “switch buttons” to control an AND gate—defining a “dual‐button‐one‐gate” mechanism.

As shown in Scheme 1, our engineered ARGON first binds specifically to PTK7 (the target of Sgc8), enabling selective entry into PTK7‐overexpressing tumor cells (e.g. HeLa) via receptor‐mediated endocytosis [51, 52, 53]. However, ARGON can also enter normal cells (e.g. HEK293T) at low levels, as PTK7 expression is not completely absent in these cells [54]. To enhance the selectivity between tumor and normal cells, the second “gate” (GSH responsiveness) is critical: elevated intracellular GSH in tumor cells cleaves the disulfide bond, removing the lock strand and unmasking ARGON's active rG4‐binding domain—while low GSH levels in normal cells minimize such activation (Scheme 1). Activated ARGON then selectively modulates rG4‐associated molecular functions and cellular activities in tumor cells, including *Bcl2* gene expression, cell migration, and proliferation. By combining spatial targeting (cell‐specific delivery) with subsequent temporal activation (GSH‐triggered switching) in an AND‐gated workflow, our study achieves programmable, precise, and spatiotemporally controlled targeting and regulation of endogenous rG4s in living cells for the first time.

## 2 Results and Discussion

### 2.1 Construct and GSH Response of ARGON

**FIGURE 1:**
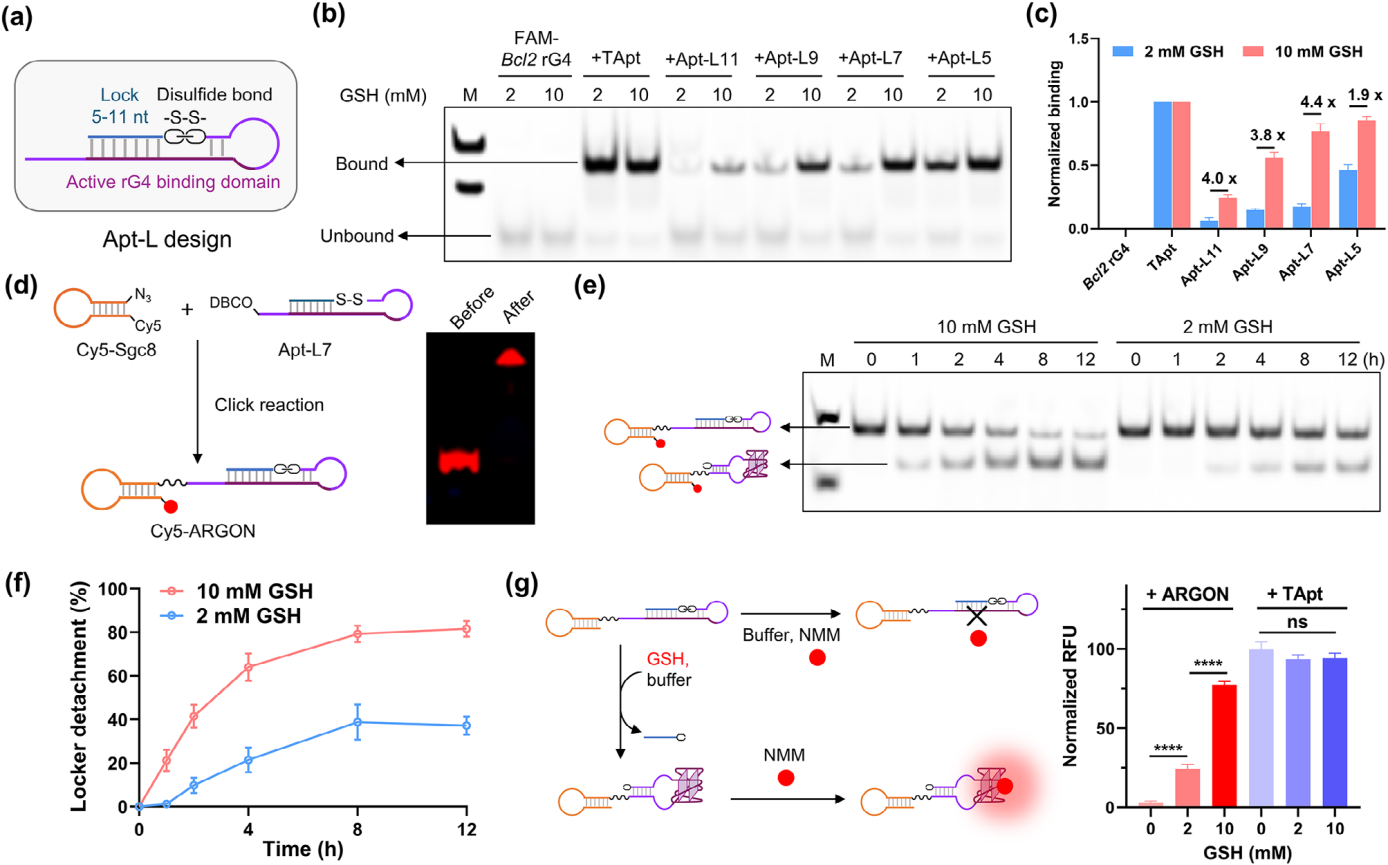
Optimization of lock strand length and construction of ARGON. (a) Design and optimization scheme of L‐RNA Apt‐L (Aptamer with Lock strand). (b) EMSA results of 400 nM TApt (Traditional Aptamer) and Apt‐L variants binding to 40 nM FAM‐*Bcl2* rG4 in binding buffer containing 2 mM or 10 mM GSH for 4 h at 37°C. “M” denotes Cy5‐labeled DNA marker, the upper band is 40 bp and the lower one is 20 bp. The gel is merged from Cy5 channel and FAM channel. (c) Quantitative analysis of FAM‐*Bcl2* rG4 binding. (d) Synthesis and PAGE analysis of Cy5‐labeled ARGON. (e) PAGE analysis of lock strand detachment from Cy5‐ARGON in binding buffer containing 2 mM or 10 mM GSH for different time at 37°C. “M” denotes Cy5‐labeled DNA marker, the upper band is 40 bp and the lower one is 20 bp. The gel was obtained from Cy5 channel. (f) Quantitative analysis of lock detachment. (g) NMM fluorescence intensity (at 615 nm emission wavelength) following incubation of TApt or ARGON with NMM in binding buffer containing different GSH concentrations. “RFU” denotes relative fluorescence units. All statistical data in are mean ± SD (*n* = 4 in (c), (f); *n* = 3 in (g)). Statistical significance: ****p < 0.0001, ns denotes no significant difference. The uncropped gels for (b) and (e) are in Supporting Information.

Prior to constructing the intact ARGON, we first identified a suitable lock strand to mask the active rG4 binding site of the parent L‐Apt.4‐1c (hereafter referred to as TApt, traditional aptamer). Our previous studies identified the G4‐forming domain within TApt as the active binding site for target rG4s (Figure S1) [22]. To generate aptamers with lock strands (Apt‐L), a complementary L‐RNA lock strand was appended to the 3’‐terminus of TApt via a disulfide bond linker. The lock strand was designed to prevent the formation of the active G4 secondary structure and unmask the active G4 domain following lock cleavage via a redox reaction. Thus, the length of the lock strand required optimization: a lock strand that is too long would result in the cleaved fragment remaining hybridized to TApt, thereby competing with target rG4s for binding; conversely, an overly short lock strand would fail to fully mask the active G4 domain, leading to unintended interactions with target rG4s even in the absence of high GSH concentrations. Therefore, as shown in Figures 1a and S2, lock strands of 5–11 nucleotides in length were appended to the 3’‐terminus of TApt via a disulfide linker to generate redox‐responsive variants Apt‐L5, Apt‐L7, Apt‐L9, and Apt‐L11, where the insertion sites are indicated by blue arrows and the secondary structures are predicted using m‐fold [55].

To identify the optimal lock length, the four Apt‐L variants and TApt (sequences provided in Table S1) were incubated with FAM‐labeled *Bcl2* rG4 in binding buffer (25 mM Tris–HCl, pH 7.5, 150 mM KCl and 1 mM MgCl_2_) supplemented with different GSH concentrations (Figure 1b). The oncogenic *Bcl2* rG4 forms in the 5’‐untranslated region (5’UTR) of *Bcl2* mRNA and regulates Bcl2 protein translation—stabilization of this rG4 by ligand modulates the expression of the anti‐apoptotic Bcl2 protein, thereby promoting cancer cell survival and inhibiting apoptosis to drive oncogenesis [56, 57]. Thus, *Bcl2* rG4 was selected as the primary oncogenic target in this study. Tumor cells typically exhibit intracellular GSH concentrations of up to 10 mM, which is 4–1000‐fold higher than those in normal cells and the extracellular environment, rendering GSH a well‐characterized TME marker [58, 59]. Herein, we used 2 and 10 mM GSH to mimic the intracellular environments of normal and tumor cells, respectively. Electrophoretic mobility shift assay (EMSA) results revealed that Apt‐L7 exhibited the greatest difference in binding to FAM‐*Bcl2* rG4 between 10 mM and 2 mM GSH (Figures 1b, c; 4.4 times). Apt‐L5 (shorter lock) showed a smaller difference in binding between GSH concentrations, despite exhibiting a slightly higher binding efficiency than Apt‐L7 in 10 mM GSH (Figure 1c). Notably, TApt showed no difference in binding efficiency between 2 mM and 10 mM GSH, indicating a lack of GSH‐dependent selectivity for targeting rG4s in tumor versus normal cell environments. Collectively, these results identified Apt‐L7 as the optimal variant for subsequent experiments.

Following the identification of the optimal Apt‐L variant (Apt‐L7), we proceeded to synthesize the full‐length ARGON via click chemistry. To facilitate the characterization of ARGON synthesis and subsequent assays, N_3_‐labeled Sgc8 was conjugated to Cy5 and reacted with DBCO‐labeled Apt‐L7 (Figure 1d). Polyacrylamide gel electrophoresis (PAGE) analysis revealed a distinct mobility shift between unconjugated and conjugated Cy5‐Sgc8 aptamer species. Additionally, mass spectrometry (MS) analysis confirmed the successful synthesis of Cy5‐ARGON (Figure S3). Its detailed information (sequence, length, cost, etc) was summarized in Tables S1 and S2. Cy5‐ARGON possess a compact composition with a total length of 73 nucleotides and a molecular weight of approximately 24.5 kDa. This concise structure minimizes synthetic reagent consumption; notably, the disulfide linkages required for redox responsiveness were incorporated directly via standard disulfide phosphoramidites during automated solid‐phase synthesis, eliminating the need for complex post‐synthetic liquid‐phase modifications. This streamlined design, combined with the modular assembly strategy, ensures that the ARGON platform remains cost‐effective and highly amenable to large‐scale manufacturing, while facilitating future sequence optimization, swapping of aptamers or locks and even displacement of disulfide chemistry for extending diverse biomedical applications.

Next, we evaluated the intrinsic redox responsiveness of Cy5‐ARGON in the absence of target rG4s. In the absence of GSH, Cy5‐ARGON remained stable, as confirmed by PAGE (Figure S4). Upon entry into cells with different intracellular GSH levels (high in tumor cells and low in normal cells), ARGON was expected to exhibit differential lock cleavage efficiency and subsequent G4 domain formation. PAGE analysis showed that 10 mM GSH induced faster and more extensive lock cleavage than 2 mM GSH (Figure 1e). The saturated lock cleavage rate in the 10 mM GSH group was approximately 2‐fold higher than that in the 2 mM GSH group (Figure 1f). To minimize premature unmasking of the active binding domain outside cells, we tested the GSH responsiveness of ARGON at 10 µM GSH, a concentration mimicking extracellular GSH levels, as reported previously [59, 60]. PAGE analysis revealed minimal lock cleavage at 10 µM GSH even after 12 h of incubation (Figure S5). These results collectively demonstrated that intracellular GSH levels in tumor cells could specifically cleave the disulfide linker and release the lock strand.

Then, we examined whether ARGON undergoes an allosteric change to reform its active G4 secondary structure following GSH‐mediated lock cleavage. N‐Methylmesoporphyrin IX (NMM), a broad‐spectrum G4 ligand that exhibits enhanced fluorescence upon binding to G4s was used to validate the proposed allosteric mechanism. To avoid potential fluorescence crosstalk, we synthesized a fluorophore‐free ARGON variant using the same click chemistry protocol (MS in Figure S6, sequence in Table S1) and measured the NMM fluorescence emission between 580 nm and 630 nm. Comparative analysis showed that incubation with high GSH concentrations resulted in a significant increase in NMM fluorescence intensity (relative to background) in the presence of ARGON (Figures S7 and 1g). Furthermore, the NMM fluorescence intensity was higher in 10 mM GSH than in 2 mM GSH. These results confirmed that intracellular GSH in tumor cells can specifically and efficiently unmask the active G4 domain by disrupting the lock strand, thereby enabling subsequent binding to the target rG4s.

### 2.2 In Vitro Characterization of ARGON Binding to *Bcl2* rG4

**FIGURE 2:**
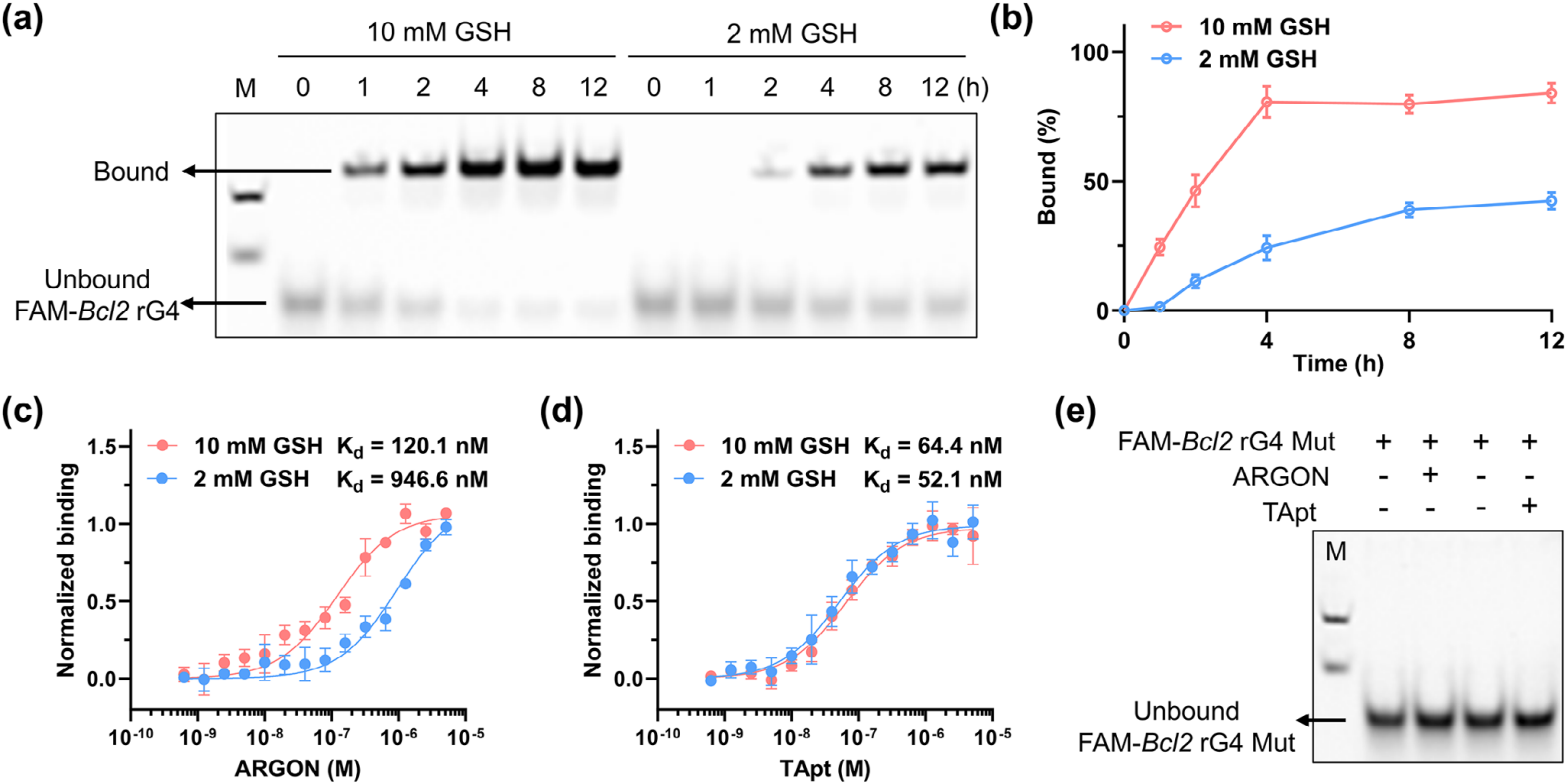
Characterization of ARGON's binding performance to rG4s. (a) Representative EMSA results for the binding kinetics of 400 nM ARGON to 40 nM FAM‐*Bcl2* rG4 in binding buffer containing 10 mM or 2 mM GSH. (b) Quantitative analysis of *Bcl2* rG4 binding kinetics. (c) MST binding curves for ARGON and FAM‐*Bcl2* rG4 in binding buffer containing 10 mM or 2 mM GSH. (d) MST binding curve for TApt and FAM‐*Bcl2* rG4 in binding buffer containing 10 mM or 2 mM GSH. (e) EMSA results for 40 nM FAM‐*Bcl2* rG4 Mut (G‐tetrad Mutant) binding to 400 nM ARGON or TApt in binding buffer containing 10 mM GSH for 4 h at 37°C. All statistical data are mean ± SD (*n* = 4 in (b); *n* = 3 in (c) and (d)). In (a) and (e), “M” denotes Cy5‐labeled DNA marker, the upper band is 40 bp and the lower one is 20 bp. The gel is merged from Cy5 channel and FAM channel. The uncropped gels for (a) and (e) are in Supporting Information.

We first examined ARGON's binding responsiveness to different GSH concentrations. ARGON was incubated with FAM‐*Bcl2* rG4 in binding buffer containing 10 mM or 2 mM GSH for different durations. EMSA results showed that the intensity of the FAM‐*Bcl2* rG4‐bound band was significantly higher in 10 mM GSH than in 2 mM GSH (Figure 2a). The saturated bound rate of FAM‐*Bcl2* rG4 was 81% in 10 mM GSH (at 4 h) versus 39% in 2 mM GSH (at 8 h) (Figure 2b). Notably, ARGON showed no binding to FAM‐*Bcl2* rG4 in the absence of GSH (Figure S8), confirming that the lock strand effectively prevented unintended binding in non‐GSH environments. Collectively, these rG4 binding results are consistent with earlier observations of lock cleavage efficiency and G4 domain formation.

Mechanistically, the ARGON activation relied on a cleavage‐induced thermodynamic switch within the Apt‐L7 module. In the OFF state, as predicted by mfold at 37°C (Figure S2), the Apt‐L7 formed a stable intramolecular hairpin (9‐bp stem) with a calculated Gibbs free energy of −15.40 kcal/mol. GSH‐mediated cleavage of the disulfide linker truncated the lock strand to a 7‐nt fragment. DINAMelt simulation indicated that the melting temperature (*T*_m_) of the resulting 7‐nt duplex was well below physiological conditions (*T*_m_ < 37°C), leading to a negligible fraction of hybridized species (∼5%) at 37°C (Figure S9) [61]. Consequently, the lock fragment spontaneously dissociated. This process was further facilitated by the target binding, where the rG4 target competitively displaced the weakly hybridized lock strand to form the stable binding complexes. And the cleavage event converted a single covalently constrained molecule into two independent species, resulting in a significant gain in translational entropy. These factors collectively drove the system to equilibrate towards the thermodynamically favored ON state.

After validating GSH‐responsive binding, we quantified ARGON's binding affinity to FAM‐*Bcl2* rG4. 40 nM FAM‐*Bcl2* rG4 was incubated with varying concentrations of ARGON (or TApt, as a control) in binding buffer containing 10 mM or 2 mM GSH, and microscale thermophoresis (MST) was used to measure the dissociation constants (*K*_d_). The binding affinity of ARGON in 10 mM GSH was 7.9‐fold that in 2 mM GSH (Kd = 120.1 nM vs. 946 nM; Figure 2c). In contrast, TApt showed similar binding affinities in 10 mM and 2 mM GSH (*K*_d_ = 64.4 nM vs. 52.1 nM; Figure 2d). Besides, the D‐type TApt (D‐Apt.4‐1c) showed virtually no binding signal under both conditions (Figure S10) due to chirality and conformational mismatch with the same rG4 target. This control confirms that the L‐RNA configuration is essential for molecular recognition in the ARGON system, in addition to the added biostability. These results highlight the critical role of conditional activation in endowing L‐RNA aptamers with selective binding to tumor‐cell rG4s.

Next, we investigated whether modifying TApt to ARGON altered its selective binding to *Bcl2* rG4 over other G4s and nucleic acid motifs. FAM‐labeled *Bcl2* rG4 Mut, a variant with mutations in the G‐tetrad region (sequence in Table S1) was used as a non‐target control. Similar to TApt, ARGON showed no binding to *Bcl2* rG4 Mut, even when its active domain was unmasked in 10 mM GSH (Figure 2e). Additionally, neither TApt nor ARGON bound to other DNA G4s (dG4s) or non‐G4 nucleic acid motifs (e.g. poly(rA), poly(rU), poly(rC), and RNA hairpins; Figure S11). To further confirm the specificity of the L‐aptamer module, we mutated the G‐tetrad region of L‐Apt.4‐1c (wildtype) and synthesized Cy5‐labeled ARGON Mut (ARGON G‐tetrad Mutant) using the same click chemistry protocol (MS in Figure S12, sequence in Table S1). EMSA results confirmed that neither TApt Mut nor ARGON Mut bound to the FAM‐*Bcl2* rG4 (Figure S13). These data collectively demonstrate that ARGON retains the selective binding of TApt to target *Bcl2* rG4 over non‐target nucleic acid structures.

### 2.3 Cellular Characterization of Access to “AND”‐Gated *Bcl2* rG4

**FIGURE 3:**
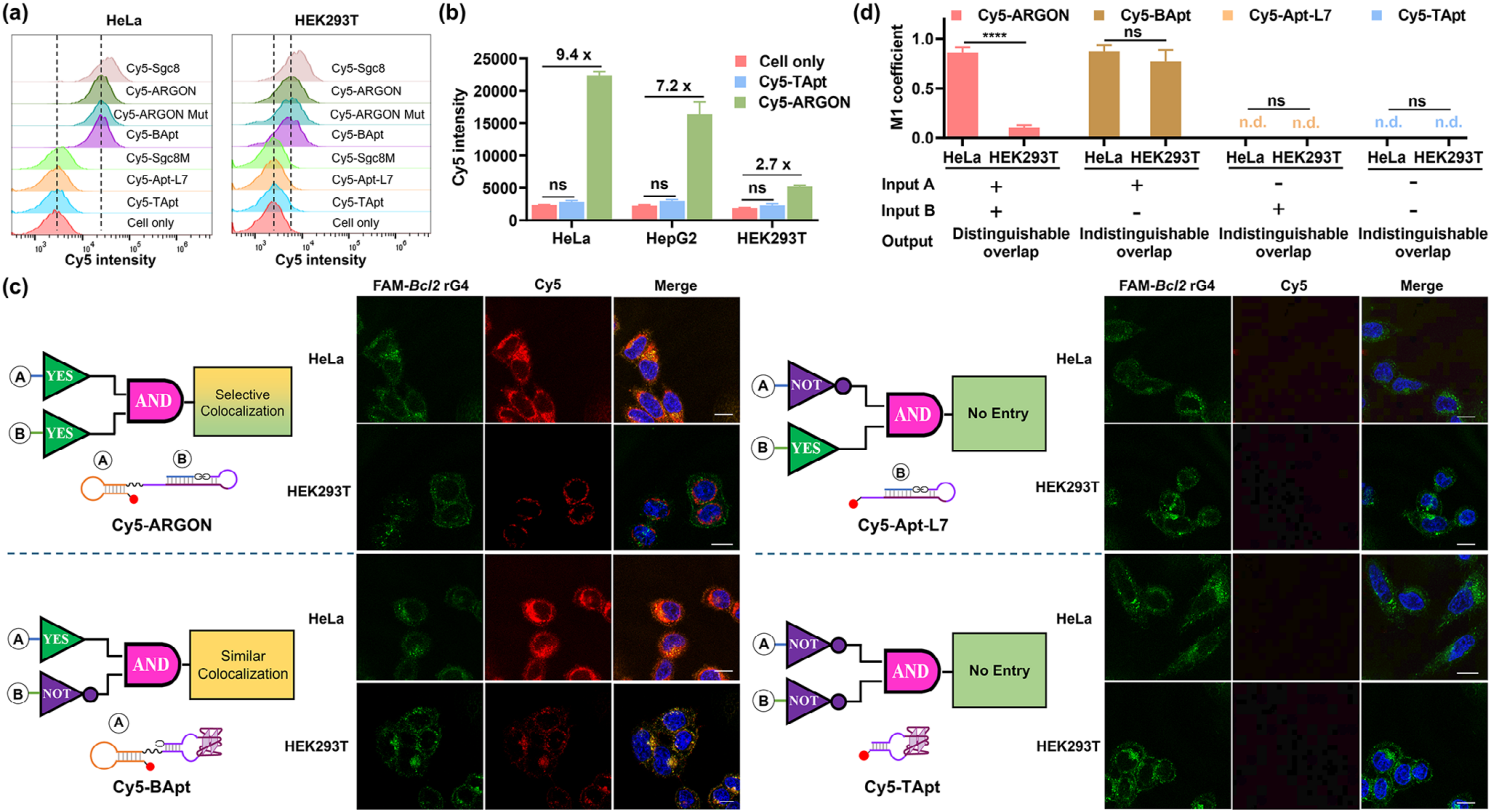
Selective internalization and rG4 targeting of ARGON in tumor cells. (a) Representative flow cytometry internalization profiles of Cy5‐ARGON and control molecules incubating with different cell lines for 4 h. ARGON Mut (ARGON G‐tetrad mutant), BApt (Bispecific Aptamer composed of TApt and Sgc8), Sgc8M (Sgc8 Mutant). (b) Quantitative analysis of cellular Cy5 fluorescence intensity after incubating with different molecules. (c) Confocal images and AND‐logic analysis of HeLa and HEK293T cells incubated with Cy5‐ARGON or control molecules for 4 h. The cells were transfected with FAM‐*Bcl2* rG4 prior to incubation. The final fields are merged with Hoechst 33342‐stained nucleus. Scale bars = 10 µm. (d) Manders' M1 coefficient (fraction of FAM‐positive pixels overlapping with Cy5‐positive pixels) for colocalization analysis under AND‐logic control. All statistical data are mean ± SD (*n* = 3). Statistical significance: ****p < 0.0001, ns denotes no significant difference. n.d. denotes not detected.

ARGON's selective targeting to tumor cell rG4s requires two “gates” (inputs): (1) cell‐specific entry via Sgc8‐PTK7 interaction, and (2) intracellular activation via GSH‐mediated lock cleavage. To validate this AND‐logic mechanism, we prepared several control molecules: Cy5‐labeled BApt (a bivalent aptamer comprising Cy5‐Sgc8 and L‐Apt.4‐1c, i.e., ARGON without the lock strand; MS in Figure S14, sequence in Table S1), Cy5‐ARGON Mut, Cy5‐Sgc8M (Sgc8 mutant), Cy5‐Apt‐L7 (Apt‐L7 without Sgc8), and Cy5‐TApt (TApt without Sgc8 or lock). We first evaluated ARGON's cell‐specific internalization via flow cytometry. HeLa and HepG2 cells (PTK7‐overexpressing tumor cells) and HEK293T cells (low PTK7‐expressing normal cells) were used as models [51, 54]. Cy5‐labeled molecules were incubated with cells without transfection, and the intracellular Cy5 fluorescence was quantified. Cy5‐ARGON, Cy5‐ARGON Mut, and Cy5‐BApt exhibited similar internalization efficiencies in each positive cell line, which is expected as they all contained the Sgc8 aptamer (Figures 3a and S15). In contrast, Cy5‐Sgc8M (mutated Sgc8) showed minimal internalization, even in PTK7‐positive HeLa and HepG2 cells. Cy5‐Apt‐L7 and Cy5‐TApt (lacking Sgc8) failed to enter any cell type without transfection. Quantitative analysis showed that intracellular ARGON levels were 2.5‐fold and 1.7‐fold higher in HeLa and HepG2 cells, respectively, than in HEK293T cells (Figure 3b), consistent with PTK7 expression levels across the three cell lines (Figure S16). HeLa (highest ARGON uptake) and HEK293T cells (low PTK7 expression) were selected for subsequent experiments. Notably, HEK293T cells still express low levels of PTK7, reflecting the non‐ideal nature of dual biomarkers in real biological systems. This underscores the necessity of the second gate (GSH‐responsive activation) to further distinguish tumor and normal cells, validating the rational design of the AND gate.

We then characterized the intracellular delivery efficiency and dose‐localization relationships of Cy5‐labeled ARGON in HeLa cells. Flow cytometry revealed a dose‐dependent uptake that scaled linearly from 50 nM to 1 µM before reaching saturation at approximately 2 µM (Figure S17). Confocal microscopy provided critical insights into the subcellular fate of the internalized probes. While ARGON predominantly co‐localized with lysosomes at low concentrations (200 nM, PCC = 0.59), increasing the dose to 5 µM significantly reduced this correlation (PCC = 0.38, Figure S18). Notably, throughout the tested concentration range, ARGON displayed negligible co‐localization with the nucleus, indicating that the probe does not accumulate in the nucleoplasm under these conditions. Instead, the observed decrease in lysosomal association at higher doses signifies a partial overflow or escape from endo‐lysosomal compartments, leading to a diffuse distribution within the cytoplasmic matrix. This cytoplasmic availability is functionally pivotal, as it positions ARGON to directly access and engage with intracellular rG4 targets residing in the cytosol.

Following validation of cell‐specific internalization (first gate), confocal microscopy was used to assess ARGON's selective colocalization with FAM‐*Bcl2* rG4 (second gate), as colocalization reflects the rG4 binding efficiency. HeLa cells transfected with FAM‐*Bcl2* rG4 were incubated with Cy5‐ARGON or Cy5‐ARGON Mut. While both conjugates exhibited similar internalization efficiencies, they showed distinct colocalization levels with FAM‐*Bcl2* rG4, as evident from the overlay images and Pearson correlation coefficients (Figure 3c, top left panel; Figure S19), consistent with their differential binding abilities (Figures 2a and S13).

We further quantified AND logic‐dependent colocalization (i.e., rG4 targeting specificity) using confocal imaging. Two inputs were defined: Module A (Sgc8 aptamer for cell entry) and Module B (lock strand for GSH activation). A positive output was defined as the differential colocalization between HeLa and HEK293T cells. When both modules were present (molecule: Cy5‐ARGON), the colocalization level of Cy5‐ARGON with FAM‐*Bcl2* rG4 was significantly higher in HeLa cells than in HEK293T cells (Figures 3c and S19b). Manders' M1 coefficient analysis showed that ∼86% of FAM‐*Bcl2* rG4 overlapped with Cy5‐ARGON in HeLa cells, compared to only 10% in HEK293T cells (Figure 3d). When only Module A was present (molecule: Cy5‐BApt, no lock strand), colocalization levels with FAM‐*Bcl2* rG4 were similar in HeLa and HEK293T cells (Figures 3c and S19b), likely because Cy5‐BApt is constitutively active and intracellular concentrations exceeded FAM‐*Bcl2* rG4 levels (Figure 3d). In the absence of Module A (Cy5‐Apt‐L7 or Cy5‐TApt), no cellular entry or colocalization with intracellular FAM‐*Bcl2* rG4 was observed. As shown in Figure S20 and concluded in Table S3, the logical performance of the ARGON system was quantified using the rG4 binding selectivity ratio (the colocalization difference between target and non‐target cells), where a ratio of ∼1 indicates non‐specific binding (OFF state) and a significantly higher ratio than 1 indicates specific target recognition (ON state). The maximal leakage is from the presence of Sgc8 alone, which yielded a selectivity ratio of 1.1, still indistinguishable from the baseline (ratio 1.0). In stark contrast, the simultaneous presence of both inputs triggered a sharp, digital transition to a ratio of 8.6. This results in a robust logic gain of approximately 7.8 (8.6/1.1), confirming that ARGON functions as a high‐fidelity digital gate capable of precise target identification with minimal background noise.

The different results in the four groups of experiments were because there were two “gatekeepers” under dual module inputs, Sgc8‐PTK7 interaction and GSH‐lock interaction, to control the gate access to selectively target tumor cellular rG4s. If one or even two gatekeepers are lacking, selective targeting or molecule access cannot be realized. Notably, ARGON without Sgc8 (i.e., Apt‐L7) or without a lock strand (i.e., BApt) is estimated to show less access or specificity to tumor cellular *Bcl2* rG4, respectively, revealing the superiority and necessity of the proposed “AND‐Logic‐Gated” chimera design with a dual molecule module design (Sgc8 + lock strand).

### 2.4 Extension of and Gated Targeting to *hTERC* rG4

**FIGURE 4:**
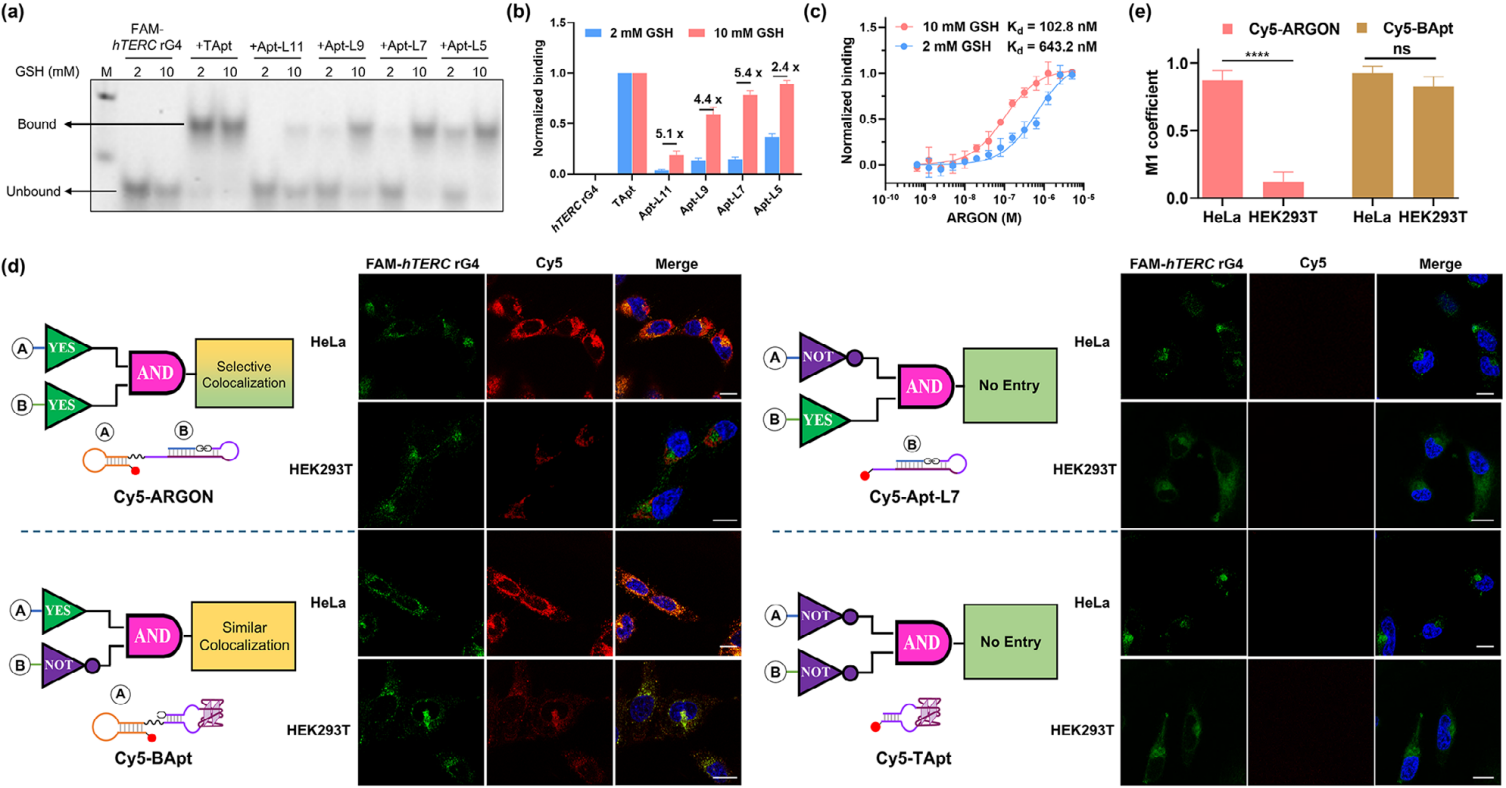
Selective binding and cellular targeting of ARGON extended to *hTERC* rG4. (a) Representative EMSA results for 400 nM TApt and Apt‐L variants binding to 40 nM FAM‐*hTERC* rG4 in binding buffer containing 2 mM or 10 mM GSH. “M” denotes Cy5‐labeled DNA marker, the upper band is 40 bp and the lower one is 20 bp. The gel is merged from Cy5 channel and FAM channel. The uncropped gel is in Supporting Information. (b) Quantitative analysis of FAM‐*hTERC* rG4 binding. (c) MST binding curves for ARGON and FAM‐*hTERC* rG4 in binding buffer containing 10 mM or 2 mM GSH. (d) Confocal images and AND‐logic analysis of HeLa and HEK293T cells incubated with Cy5‐ARGON or control molecules. The cells were transfected with FAM‐*hTERC* rG4 prior to incubation. The final fields are merged with Hoechst 33342‐stained nucleus. Scale bars = 10 µm. (e) Manders' M1 coefficient (fraction of FAM‐positive pixels overlapping with Cy5‐positive pixels). All statistical data are mean ± SD (*n* = 4 in (b); *n* = 3 in (c) and (e)). Statistical significance: ****p < 0.0001, ns denotes no significant difference. n.d. denotes not detected.

Encouraged by ARGON's selective targeting of *Bcl2* rG4, we extended this strategy to *hTERC* rG4 to present the ARGON's versatility of by continuously employing modified L‐Apt.4‐1c and Sgc8. First, we optimized the lock strand length for *hTERC* rG4 targeting. Different Apt‐L variants were incubated with FAM‐*hTERC* rG4 in binding buffer containing 10 mM or 2 mM GSH (sequences in Table S1). EMSA results showed that Apt‐L7 exhibited the largest difference in binding to FAM‐*hTERC* rG4 between 10 and 2 mM GSH (Figure 4a, b), consistent with the *Bcl2* rG4 targeting results. Thus, the same ARGON conjugate (synthesized previously) was used for *hTERC* rG4 targeting.

Next, we characterized ARGON's binding to *hTERC* rG4. Kinetic EMSA showed that the saturated bound fraction of FAM‐*hTERC* rG4 was 84% in 10 mM GSH (4 h) versus 37% in 2 mM GSH (8 h; Figure S21). For sequence specificity, neither ARGON nor TApt bound to FAM‐*hTERC* rG4 Mut (G‐tetrad mutant; Figure S22). MST analysis showed that ARGON exhibited ∼5.3‐fold higher binding affinity to FAM‐*hTERC* rG4 in 10 mM GSH than in 2 mM GSH (*K*_d_ = 102.8 nM vs. 643.2 nM; Figure 4c), whereas TApt showed similar affinities under both conditions (*K*_d_ = 60.9 nM vs. 47.2 nM; Figure S23). These kinetic and dose‐dependent data confirm that ARGON binds *hTERC* rG4 faster, more efficiently, and with higher affinity in tumor‐mimetic (10 mM GSH) than in normal‐mimetic (2 mM GSH) environments.

After validating the in vitro GSH‐responsive binding, we assessed ARGON's cellular targeting of *hTERC* rG4 under AND logic control. When both Sgc8 and the disulfide‐tethered lock were present (molecule: Cy5‐ARGON), colocalization with FAM‐*hTERC* rG4 was significantly higher in HeLa cells than in HEK293T cells (Figures 4d and S24). Manders' M1 analysis showed that approximately 87% of FAM‐*hTERC* rG4 overlapped with Cy5‐ARGON in HeLa cells, compared to only 12% in HEK293T cells (Figure 4e). In contrast, Cy5‐BApt (lacking the lock strand) showed similar colocalization levels with FAM‐*hTERC* rG4 in both cell lines (Figures 4e and S24). As observed with *Bcl2* rG4, Cy5‐Apt‐L7, and Cy5‐TApt (lacking Sgc8) failed to enter the cells or colocalize with intracellular FAM‐*hTERC* rG4 (Figure 4d). These results highlight again that the dual‐input AND gate (Sgc8‐PTK7 interaction + GSH‐lock cleavage) enables the selective targeting of specified rG4s of interest within tumor cells.

### 2.5 Selective Functional Regulation of rG4‐Linked Activities

**FIGURE 5:**
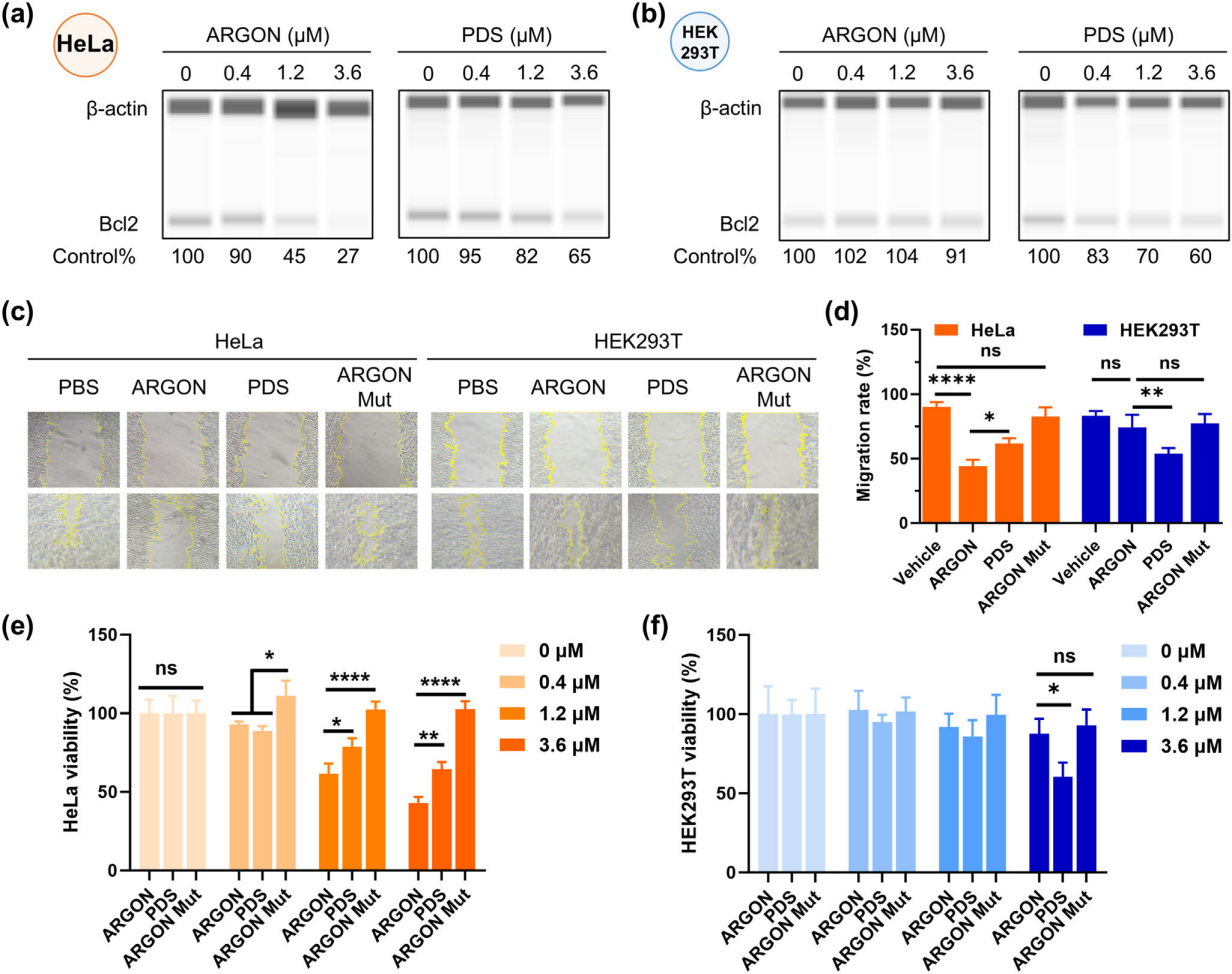
Selective regulation of rG4‐linked downstream cellular activities. Capillary electrophoresis‐based Simple Western analysis of (a) HeLa and (b) HEK293T cells following incubation with varying concentrations of ARGON and PDS (pyridostatin) for 48 h. (c) Wound closure images of HeLa and HEK293T cells treated with 3.6 µM ARGON or other control molecules for 48 h. (d) Quantitative analysis of wound closure rates. (e) Viability of HeLa cells following treatment with varying concentrations of molecules for 72 h. (f) Viability of HEK293T cells following treatment with varying concentrations of molecules for 72 h. All statistical data are mean ± SD (*n* = 3). Statistical significance: ****p < 0.0001, **p < 0.01, *p < 0.05, ns denotes no significant difference. The uncropped blots for (a) and (b) are in Supporting Information.

Following the validation of the AND‐logic targeting mechanism, we investigated ARGON's ability to modulate rG4‐associated functions and cellular activities. First, we evaluated ARGON's effect on Bcl2 protein expression in HeLa and HEK293T cells as it was reported that L‐aptamers can inhibit gene expression by stabilizing related rG4s [18, 32]. Cells were incubated with varying concentrations of ARGON (or controls) for 48 h, and Bcl2 protein levels were measured using capillary electrophoresis‐based Simple Western analysis. ARGON downregulated Bcl2 protein expression to 27% of the control level at 3.6 µM, whereas ARGON Mut (non‐binding variant) had no effect (Figures 5a and S25a). As a positive control, pyridostatin (PDS), a broad‐spectrum G4 stabilizer, also downregulated Bcl2 protein expression in HeLa cells, but with lower efficiency than ARGON (Figure 5a). Notably, PDS inhibited Bcl2 expression to 60% of the control level in HEK293T cells at 3.6 µM (Figure 5b), whereas ARGON had no significant effect on HEK293T cell Bcl2 expression (Figures 5b and S25b). This stark contrast arises because PDS is non‐selectively internalized into all cell types and exhibits constitutive G4‐binding activity, whereas ARGON's AND‐logic design restricts activation to tumor cells. To clarify the mechanism of Bcl2 downregulation, we quantified *Bcl2* mRNA levels via reverse‐transcription quantitative real‐time polymerase chain reaction (RT‐qPCR), and no significant changes were observed in either HeLa or HEK293T cells following treatment with ARGON or controls (Figure S26). This confirms that ARGON inhibits Bcl2 protein expression at the translational level by stabilizing *Bcl2* rG4 (a known repressor of *Bcl2* mRNA translation) rather than modulating transcriptional activity.

We then assessed ARGON's impact on cell migration—a process promoted by oncogenic *Bcl2*. A wound‐healing assay was performed as follows: HeLa and HEK293T cells were scratched to create a wound and then treated with PBS vehicle, 3.6 µM ARGON, PDS or ARGON Mut for 48 h. In HeLa cells, the wound closure rate in the ARGON group was 44% compared to 90% in the PBS vehicle group and 62% in the PDS group (Figure 5c, d), demonstrating ARGON's potent migration inhibition to tumor cells. And ARGON Mut group did not show difference with vehicle group, consistent with previous no binding results between ARGON Mut and *Bcl2* rG4 (Figures S13 and S19). In contrast, ARGON and ARGON Mut had no significant intervention on normal wound closure in HEK293T cells while PDS displayed apparent inhibition to HEK293T cellular normal migration (Figure 5c, d), confirming that ARGON had cell type‐specific activity over than small molecule ligand, PDS.

Given that overexpressed Bcl2 protein supports unlimited tumor cell proliferation, we evaluated ARGON's effect on cell viability. ARGON inhibited HeLa and HepG2 cell proliferation in a dose‐dependent manner, with significant suppression observed at 1.2 µM (Figures 5e and S27). Importantly, ARGON had no impact on HEK293T cell viability across all tested concentrations (Figure 5f). ARGON Mut also failed to affect proliferation in any cell type, ruling out non‐specific cytotoxicity. In comparison, PDS inhibited tumor cell proliferation but also induced cytotoxicity in HEK293T cells at 3.6 µM (Figure 5f), further highlighting ARGON's superior safety profile, driven by its AND‐logic‐controlled activation.

While this study demonstrated the regulation potential of ARGON, we also noticed that the cellular imaging models utilized transfected rG4 reporters, which may not perfectly mimic the steric and kinetic constraints of endogenous ribonucleoprotein complexes. Nevertheless, the observed regulation of endogenous Bcl2 expression and cellular phenotypes confirmed the platform's efficacy against native targets. Future work will focus on validating these interactions in endogenous and physiological‐relevant models, to further bridge the gap between simplified imaging proxies and complex realistic environments.

Building on this successful extension to the *hTERC* rG4, it is important to recognize that while the AND‐gated design principle of ARGON is potentially extendable and generalizable, the vast structural diversity of rG4s necessitates the use of distinct L‐aptamer variants within the ARGON framework to specifically recognize each target class. Since the lock strand functions by sequestering the active binding domain of the L‐aptamer rather than the target itself, the specific sequence, length, and stability of this locking interaction must be empirically tuned for each unique pair of rG4 target and L‐aptamer. This tuning is critical to reconcile two competing requirements: (i) ensuring sufficient stability of the locked state to minimize off‐target binding in the absence of the trigger (or at low concentrations), and (ii) enabling high unlock efficiency to achieve maximal activation and high‐affinity binding upon specific trigger recognition. Of note, the ‘generality’ of our ARGON refers to its universal logic architecture—the AND‐gated heterodimeric design that decouples recognition from activation—rather than implying a universal sequence construct or a one‐size‐fits‐all solution capable of recognizing all rG4 targets or distinguishing all malignancies from normal tissues without customization. Instead, successful application will require context‐specific optimization of input markers (‘tailored design’) to match the unique molecular signature of each disease type.

Despite the promising specificity demonstrated by ARGON, there is still the inherent limitations of using GSH as a sole metabolic trigger. Moderate or fluctuating GSH concentrations, capable of reducing the disulfide lock, are present in certain non‐malignant tissues (e.g., liver), posing a potential risk for off‐target activation in clinical translation. The current safety profile of ARGON relies critically on its dual‐key AND‐gate architecture: effective regulation requires the coincidence of intracellular GSH‐mediated unlocking and high‐density PTK7‐mediated cellular uptake. This ‘two‐factor authentication’ significantly narrows the window for false positives, as healthy tissues rarely co‐express both markers at threshold levels. Nevertheless, to further mitigate risks in complex physiological environments, future iterations of this platform should evolve towards multi‐input logic systems (e.g., three‐input AND gates) that integrate orthogonal triggers such as reactive oxygen species (ROS) or tumor‐specific proteases.

## 3 Conclusion

In this study, we have reported the rational design of ARGON, a first AND‐logic‐gated allosteric heterodimeric aptamer, for precise targeting and modulation of oncogenic rG4s in tumor cells. By integrating two modular components (Sgc8 DNA aptamer for PTK7‐mediated cell entry and GSH‐cleavable lock‐masked L‐Apt.4‐1c for rG4 binding), ARGON achieves tumor‐specific activation: it enters PTK7‐overexpressing tumor cells via receptor‐mediated endocytosis and selectively unlocks its rG4‐binding activity in the high‐GSH TME. This dual‐input control ensures selective binding to tumor cellular rG4s (*Bcl2* rG4 and *hTERC* rG4 as proof of concept) and modulation of downstream cellular functions (Bcl2 protein expression, cell migration, and proliferation) while sparing normal cells from being affected. Rigorous validation with lock‐deficient (BApt) and Sgc8‐deficient (Apt‐L7, TApt) controls confirms that both modules are indispensable for precise tumor targeting—addressing the longstanding challenge of off‐target rG4 modulation in normal cells. Compared to broad‐spectrum G4 ligands (e.g. PDS), ARGON exhibits superior specificity and safety, which can be attributed to its programmable logic‐gated design and specific interactions. To the best of our knowledge, this study represents the first demonstration of a chimeric AND‐gate device that couples a tumor‐targeting D‐DNA aptamer with a conditionally activated L‐RNA aptamer, enabling precise intracellular targeting and functional modulation of rG4s.

Collectively, our study establishes ARGON as a plug‐and‐play molecular platform that unites cell‐surface addressing, intracellular stimulus sensing and RNA‐structure‐directed regulation within a single AND‐gated device. We anticipate that this logic‐gated architecture will inspire future efforts to construct intelligent nanodevices capable of integrating orthogonal recognition events, performing in‐cell computation, and executing regulatory decisions only when complex biological signatures are satisfied. It is crucial to emphasize that this work establishes a smart platform concept; substantial further optimization, including pharmacokinetic profiling, immunogenicity assessment, and extensive preclinical studies, is required before any potential clinical translation can be considered. Thus, this study pushes the boundaries of nucleic acid‐based precision research tools towards more advanced biological applications.

## Author Contributions

**Chun Kit Kwok**: conceptualization, methodology, formal analysis, supervision, funding acquisition, visualization, resources, project administration, writing – review and editing, validation. **Ying Feng**: software, data curation, visualization, validation. **Dan Wang**: conceptualization, methodology, funding acquisition, investigation, validation, formal analysis, data curation, visualization, project administration, writing – original draft, writing – review and editing, software, resources.

## Conflicts of Interest

The authors declare no conflicts of interest.

## Supporting information




**Supporting File**: Supporting Information is available on the *Angewandte Chemie* website.

## Data Availability

The data that support the findings of this study are available from the corresponding author upon reasonable request.
