# Supplementary material for: AND‐Logic‐Gated Aptamer Switch for Precise Targeting and Regulation of RNA G‐Quadruplexes
Source: Angew Chem Int Ed Engl. 2026 May 13;65(27):e4594145. doi: 10.1002/anie.4594145 (PMC13327631; doi:10.1002/anie.4594145)
Supplement: Supplementary file 1 — Supporting File: Supporting Information is available on the Angewandte Chemie website. [file ANIE-65-e4594145-s001.pdf]

Supporting Information

AND-Logic-Gated Aptamer Switch for Precise Targeting and Regulation of RNA G-Quadruplexes

Dan Wang[a], Ying Feng[a], Chun Kit Kwok*[,a,b]

[a]Department of Chemistry and State Key Laboratory of Marine Environmental Health, City University of Hong Kong, Tat Chee Avenue, Kowloon Tong Hong Kong SAR 999077, China

[b]Shenzhen Research Institute of City University of Hong Kong, Shenzhen 518057, China.

*Correspondence: ckkwok42@cityu.edu.hk

Outline

1. Materials and Methods 3

2. Supporting Figures 7

3. Supporting Tables 35

4. References 40

1. Materials and Methods

General materials

Glutathione (GSH) and other small molecules were purchased from J&K Chemical and Energy Chemical unless specified. All nucleic acid oligonucleotides were obtained from Hippo Biotech and Genewiz Biotech, unless homemade in the lab. For structure refolding, all oligonucleotides were heated at 95 °C for 5 min, then at 25 °C for at least 5 min in binding buffer before use. Zymo-Spin IC Column used for conjugates purification was obtained from ZYMO Research. Acrylamide-Bis-acrylamide and Lipofectamine® 2000 were purchased from Thermofisher and Invitrogen. RNeasy Plus Mini Kit was obtained from Qiagen. Bovine serum albumin (BSA) was obtained from Beyotime Biotech. All primary and secondary antibodies were purchased from Proteintech and Thermofisher. Simple Western™ consumables were purchased from ProteinSimple. 5x PrimeScript RT Master Mix and 2x SsoAdvanced Universal SYBR Green Supermix were purchased from TAKARA and Bio-Rad, respectively. Cell Counting Kit-8 (CCK-8) was purchased from New Cell & Molecular Biotech and APExBIO. Binding buffer is composed of 25 mM Tris–HCl, pH 7.5, 150 mM KCl and 1 mM $MgCl_2$. Running buffer is composed of 25 mM Tris–HCl, pH 7.5, 50 mM KOAc and 1 mM $MgCl_2$. Pasteur pipettes and all consumables for cell culture were purchased from SPL Life Sciences and NEST Biotechnology. Other reagents were obtained from Thermofisher unless otherwise specified.

Polyacrylamide gel electrophoresis (PAGE) and electrophoretic mobility shift assay (EMSA)

To optimize the length of Apt-L (aptamer with a lock strand), 40 nM FAM-*Bcl2* or FAM-*hTERC* rG4 (RNA G-quadruplex) and 400 nM different Apt-L variants or TApt (traditional aptamer) were incubated in binding buffer with 2 mM or 10 mM GSH at 37 °C for 4 h. To characterize the detachment kinetics of the lock strand, 40 nM Cy5-ARGON (Allosteric RNA G-quadruplex ON-switch) was incubated with binding buffer with different concentrations of GSH at 37 °C for different times. To test the binding kinetics between ARGON and rG4s, 40 nM FAM-rG4s and 400 nM ARGON were incubated in binding buffer with the relevant GSH concentration at 37 °C for the designed time. To investigate sequence-specific binding selectivity, 40 nM FAM-labeled rG4s or other nucleic acids were incubated with 400 nM ARGON or TApt in binding buffer with 10 mM GSH at 37 °C for 4 h. All samples were loaded onto a 10% native polyacrylamide gel and run in running buffer for suitable time. Home-made Cy5-labeled DNA marker has two bands, 40 bp and 20 bp. The gel was scanned using an Amersham Typhoon Laser-Scanner and analyzed using ImageJ. All gel images were acquired directly via fluorescence scanning in the FAM or Cy5 channels without post-electrophoretic staining. In Figure 1b, 2a, 2e and 4a, final composite figures were generated by overlaying the FAM-channel signals with the Cy5-labeled marker in Cy5 channel.

Click reaction

To synthesize the Cy5-labeled ARGON conjugate, a mixture containing 300 μM of DBCO-modified Cy5-Sgc8 and 200 μM of azide-modified Apt-L7 was prepared in phosphate-buffered saline (PBS). This reaction mixture was incubated at 37 °C with continuous shaking overnight to facilitate the copper-free click chemistry between the dibenzocyclooctyne (DBCO) and azide groups, resulting in the formation of the desired conjugate. Following the reaction, the conjugate was purified using a spin column to remove unreacted components and byproducts, ensuring a high-purity product suitable for downstream applications. Then the purified conjugate was analyzed by 10% denaturing PAGE and 4800 Plus MALDI TOF/TOF Analyzer (ABI) to validate the conjugate's purity and identity. Other heterodimeric aptamer conjugates in the study were synthesized following this same protocol to ensure consistency and reproducibility across samples.

Fluorescence measurement

To test the NMM-enhanced fluorescence, 500 nM ARGON or TApt was incubated with 2 μM NMM in binding buffer with different concentrations of GSH at 37 °C for 4 h. Then the mixture was transferred into a black 96 well plate. The emission spectra from 580 to 630 nm were collected at 399 nm excitation using SpectraMax iD5 Multi-Mode Microplate Reader. The relative fluorescence unit (RFU) at 615 nm emission was served as quantitative data.

Microscale thermophoresis (MST)

To test binding affinity using MST, 50 nM of FAM-*Bcl2* rG4 or FAM-*hTERC* rG4 were incubated with different concentrations of ARGON or TApt in binding buffer with 2 mM or 10 mM GSH at 37 °C for 4 h. After incubation, MST measurements were carried out with the NanoTemper Monolith NT.115 instrument using the "nano-blue" channel. The data obtained were analyzed using GraphPad Prism software.

Cell culture

All cell lines were purchased from ATCC (American Type Culture Collection, Manassas, VA, USA). HeLa (human cervical cancer cell), HEK293T (human embryonic kidney cell) and HepG2 (human liver cancer cell) were cultured in DMEM media supplemented with 10% fetal bovine serum, 100 U/mL penicillin and 100 μg/mL streptomycin at 37 °C in 5% CO2 atmosphere. PBS was used to wash wells. Trypsin and enzyme-free EDTA digestion reagent were used to detach cells from dishes or plates.

Flow cytometry assay

All flow cytometry measurements were performed with CytoFLEX. All cells (a total of 2 x 10^5) were seeded in a 12-well plate and incubated overnight for adherence before treatment. To

test the cell internalization efficiency, 1 μM Cy5-ARGON and other Cy5-labeled molecules were incubated with cells at 37 °C for 4 h. To measure the PTK7 expression levels, cells were blocked with 3% BSA and then incubated with rabbit anti-PTK7 antibody (1: 500) or IgG control. Then the cells were washed and stained with Alexa Fluor 633 goat anti-rabbit IgG (1: 400, Thermofisher cat. A21070). All cells were collected and washed three times to remove nonspecific attachment before testing by flow cytometry.

Confocal imaging

All confocal imaging experiments were performed with Leica TCS SPE and SP5. 5 x 10^4 cells were seeded in 15 mm confocal dishes and incubated overnight for adherence before experiments. To test the colocalization, 40 pmol FAM-*Bcl2* rG4 or FAM-*hTERC* rG4 was transferred to cells by lipofectamine for 4 h. Then the cells medium was replaced with fresh DMEM medium containing 1 μM Cy5-ARGON or other molecules. After 4 h incubation, Hoechst 33342 was used to stain cellular nuclei. Finally, cells were washed and then imaged by confocal microscope.

Capillary electrophoresis (CE) -based Simple Western™

CE-based Simple Western™ protocol was based on the instructions of the manufacturer and previous reports with some modifications.[1-2] 4 x 10^5 cells were seeded in 6-well plate and incubated overnight for adherence before experiments. Different concentrations of ARGON or other control molecules like PDS (pyridostatin) and ARGON Mut (mutant), was incubated for 48 h. Cells were harvested and then treated by RIPA buffer with 1% Protease Cocktail Inhibitor (Thermofisher) at 4 °C. Next cell lysates were incubated on ice for 15 to 30 min, followed by centrifuge at 13000 rpm at 4 °C for 20 min. Protein samples were added into the plate the manufacturer provided (ProteinSimple) to execute a CE-Western Blotting analysis by Protein Simple Instrument. Target Bcl2 protein was analyzed by rabbit anti-Bcl2 primary antibody (1: 30, Proteintech, cat. #12789-1-AP). β-actin protein as an internal reference was analyzed by rabbit anti-β-actin primary antibody (1:1500, Cell Signaling Technology). These primary antibodies are stained by HRP-labeled secondary antibody provided by manufacturer (Proteintech, cat. #DM-001). Protein bands were visualized by Compass Software and analyzed by ImageJ.

RT-qPCR (reverse-transcription quantitative real-time polymerase chain reaction) assay

2 x 10^5 cells were seeded in 12-well plate and incubated overnight for adherence before experiments. Different concentrations of ARGON or other molecules was incubated for 48 h to investigate the *Bcl2* mRNA levels. Cells were harvested and then extracted total RNA by RNeasy Plus Mini Kit (Qiagen). Then 200 ng total RNAs were reverse transcribed into cDNA using PrimeScript RT Master Mix provided by the manufacturer (Takara, cat. #RR036A). Then

the mixture was collected to perform quantitative polymerase chain reaction (qPCR) using designed primers and SsoAdvanced Universal SYBR Green Supermix provided by the manufacturer (Bio-Rad, cat. #172-5271). The qPCR primers for *Bcl2* cDNA and *GAPDH* cDNA were provided in Table S1. qPCR assay was subjected to a CFX Connect Real-Time System (Bio-Rad) under the following annealing procedure: denaturation 98 °C 30 s, denaturation 98 °C 10 s, annealing / extension 60 °C 30 s, total 40 cycles.

Cell migration assay

To evaluate the cell migration using a wound healing assay, cells were first seeded into 12-well plate at a density of $4×10^5$ cells/well and cultured until they reached 70-90% confluence to form a continuous monolayer. A sterile 200 μL pipette tip was then used to create uniform linear wounds across the cell monolayer, and the wells were gently rinsed with PBS to remove detached cells and cell debris. DMEM medium containing 3.6 μM ARGON, PDS and ARGON Mut was added to the treated wells and incubated for 48 h, while the control wells received the medium with only the PBS vehicle. The images of the wound area were captured at both 0 h (immediately after wounding) and 48 h (at the end of incubation), Finally, migration efficiency was quantified using ImageJ software by measuring the percentage of wound closure.

Cytotoxicity measurement

To test cell proliferation situations through cytotoxicity measurement, cells were seeded in a 96-well plate (5000 cells/well) and then incubated overnight for adherence. Afterwards, 200 μL DMEM medium containing different concentrations of ARGON or other molecules (3.6, 1.2, 0.4, and 0 μM) were added into cells to incubate 72 h. For CCK-8 analysis, the cell medium was replaced with 100 μL fresh cell medium containing 10% CCK-8. After incubating for the proper time, cell viability was determined by measuring absorbance at 450 nm using a SpectraMax iD5 Multi-Mode Microplate Reader.

Data statistics. For colocalization analysis of confocal images and band intensity analysis of gels and western blots, ImageJ software (https://imagej.nih.gov/ij) was used. For statistical analysis, Graphpad Prism 10.0 (https://www.graphpad.com) was used to show differences between two groups (Student’s t test) or among three or more groups (one-way analysis of variance or two-way analysis of variance). All statistical data are mean ± s.d., n = 3 unless specified. Statistical significance: ****$P < 0.0001$, ***$P < 0.001$, **$P < 0.01$, *$P < 0.05$, ns denotes no significant difference. It is an obvious difference when P value is less than 0.05.

2. Supporting Figures

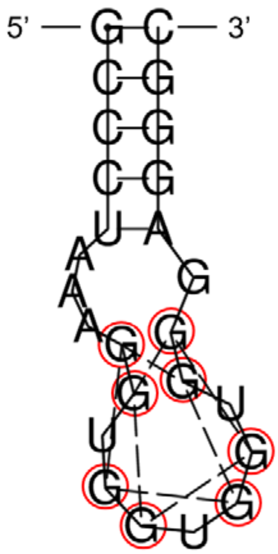

Figure S1. Predicted secondary structure of original L-Apt.4-1c using RNAfold Web Server. The G4 domain, highlighted with a red circle, represents the active binding site for target RNA G-quadruplexes (rG4s). This structure demonstrates how the active rG4 binding site can be blocked or masked by a partially complementary lock strand, as shown in Figure 1 and Figure S2, indicating the aptamer's regulated binding capability.

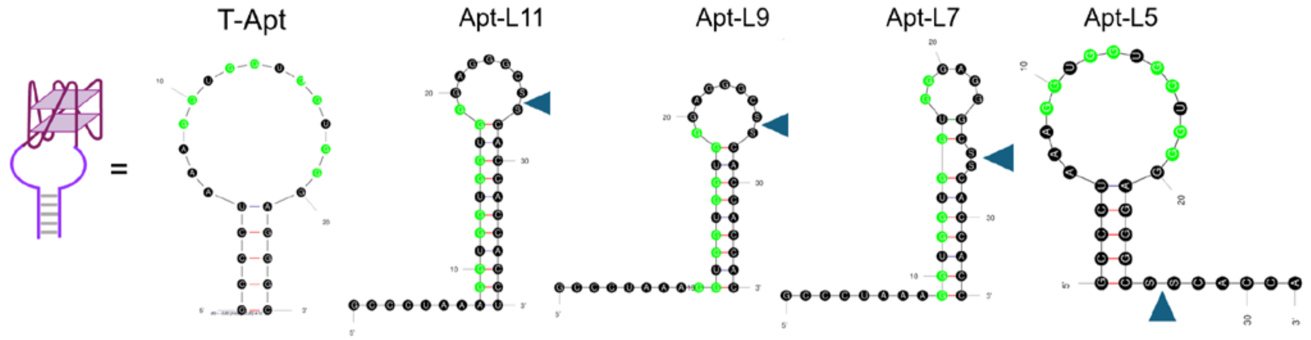

Figure S2. Predicted secondary structures of L-Apt.4-1c with different lengths of lock strands (Apt-L) using mfold.[3] Arrows indicate the disulfide bond positions. Each predicted structure represents the sole or preferential conformation of the respective Apt-L variant, illustrating varying extents of unmasking of the active rG4 binding domain, which affects the aptamer's activation.

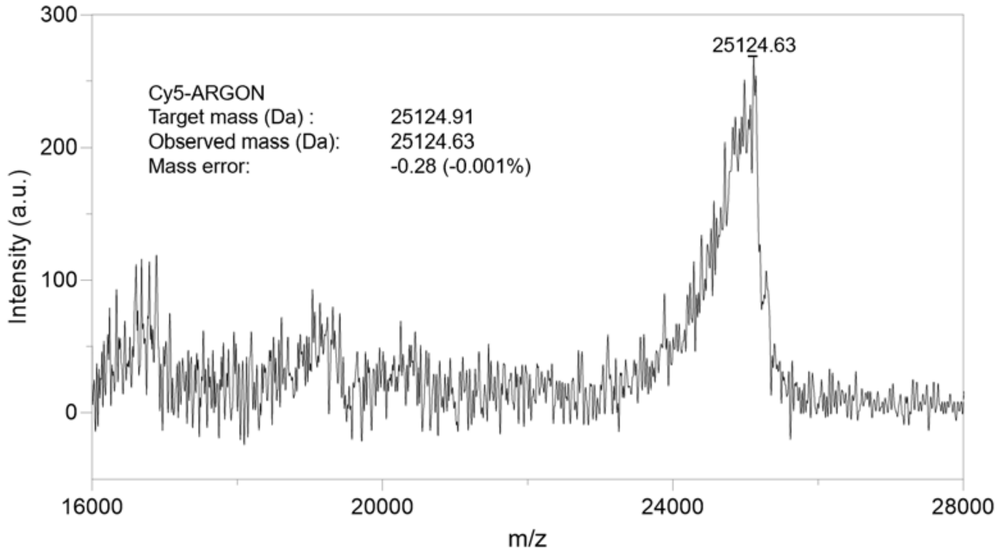

Figure S3. Mass spectra result of Cy5-labeled ARGON by MALDI-TOF assay. Mass calculated: 25124.91, mass found: 25124.63. This suggests that Cy5-ARGON was synthesized successfully.

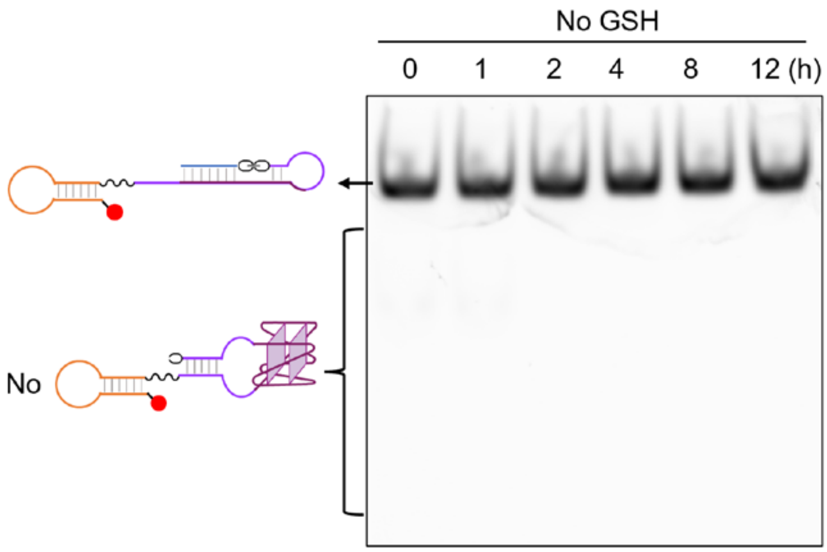

Figure S4. Stability of Cy5-ARGON in the absence of GSH. Cy5-ARGON was incubated in binding buffer without GSH at 37 °C for various durations and analyzed by native PAGE. The results show a single, stable band over time, demonstrating that ARGON does not degrade or undergo significant conformational change under these non-reducing conditions.

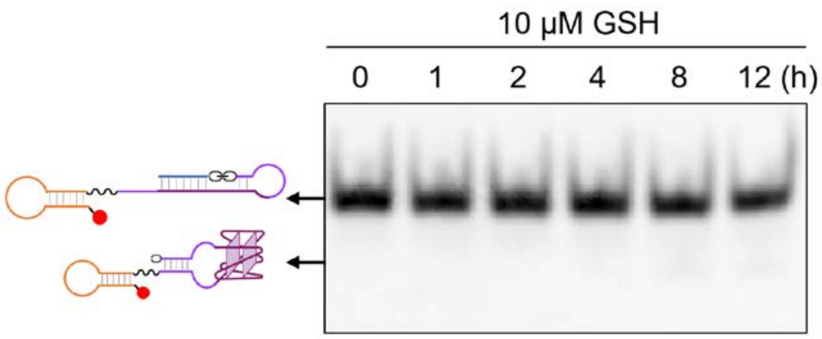

Figure S5. Lock strand detachment analysis under extracellular GSH conditions. Cy5-ARGON was incubated in binding buffer with 10 μM GSH, mimicking extracellular concentrations, and analyzed by PAGE. No significant lock detachment was observed, confirming ARGON's stability and minimal premature activation in the extracellular environment.

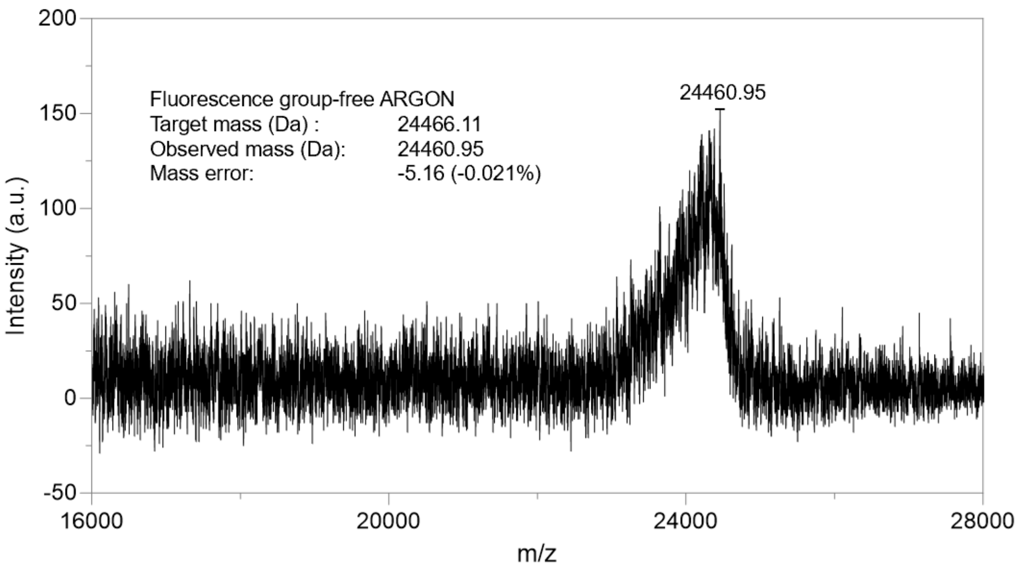

Figure S6. Mass spectra result of fluorescence group-free ARGON by MALDI-TOF assay. Mass calculated: 24466.11, mass found: 24460.95. This suggests that ARGON was synthesized successfully.

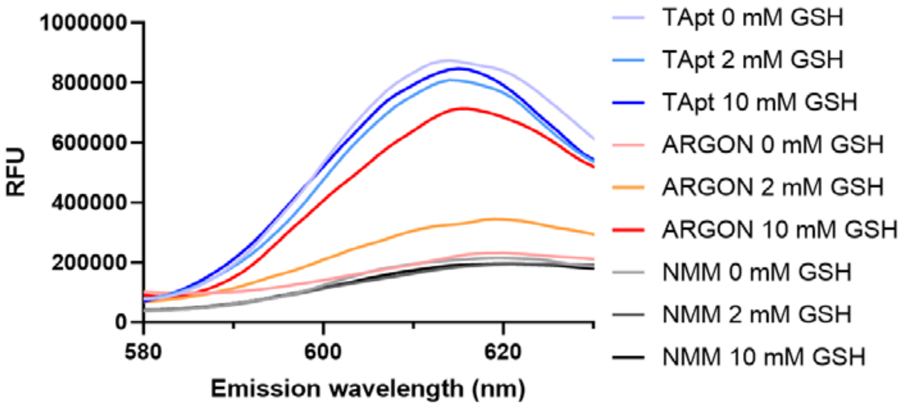

Figure S7. NMM fluorescence emission spectra for G4 formation analysis. Spectra were recorded before and after incubating TApt or ARGON with NMM in binding buffer containing different GSH concentrations at 37 °C for 4 h. NMM-enhanced fluorescence indicates the presence of G-quadruplex structures recognized by the NMM ligand, reflecting the folding state of the aptamers under various redox conditions.

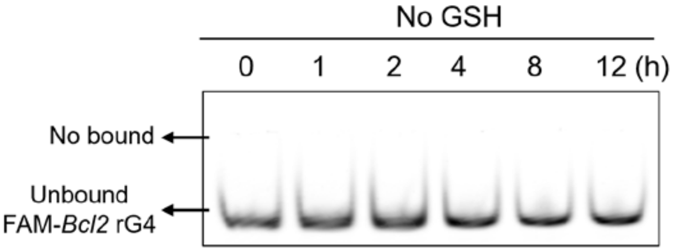

Figure S8. Binding kinetics of ARGON to FAM-*Bcl2* rG4 in the absence of GSH. A mixture of 400 nM ARGON and 40 nM FAM-*Bcl2* rG4 was incubated in GSH-free binding buffer for up to 12 h and analyzed by EMSA. The absence of a shifted band across all time points confirms that ARGON remains inactive and does not bind its target without the reducing trigger, preventing false-positive leakage.

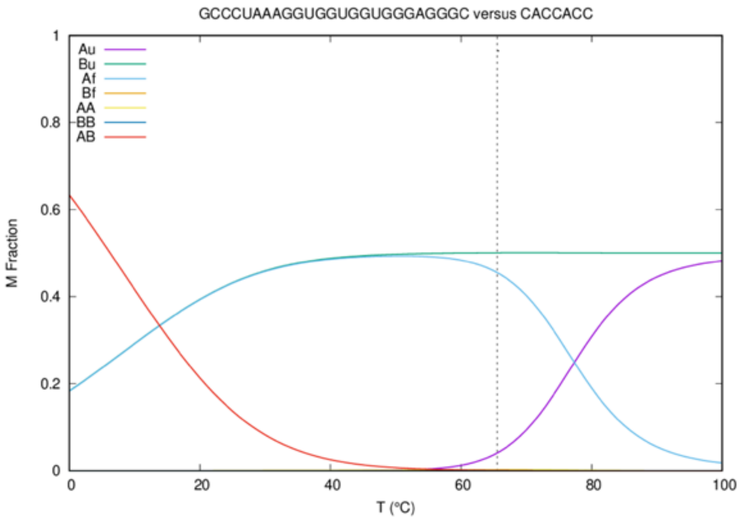

Figure S9. Thermodynamic hybridization simulation generated by DINAMelt.[4] In the simulation, strand A represents the TApt and strand B represents the 7-nt lock strand. The red curve (AB) indicates the molar fraction of the duplex complex formed between A and B. The results demonstrate a very low melting temperature (T_m) for the AB complex, with a residual duplex fraction of only approximately 5% at 37°C, indicating that the TApt and lock strand largely dissociate under physiological conditions.

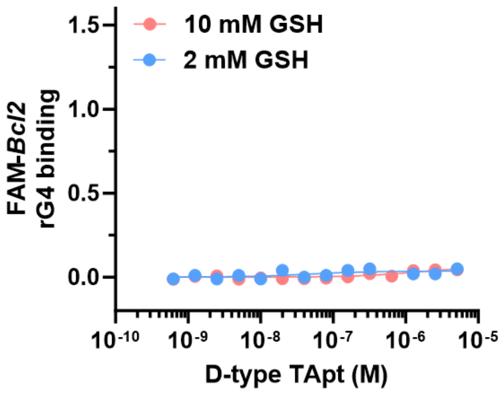

Figure S10. MST binding curve for D-type TApt (D-Apt.4-1c) and FAM-*Bcl2* rG4 in binding buffer containing 10 mM or 2 mM GSH. The D-type aptamer shows no binding signal due to stereochemical mismatch, confirming the necessity of the L-RNA configuration for ARGON function.

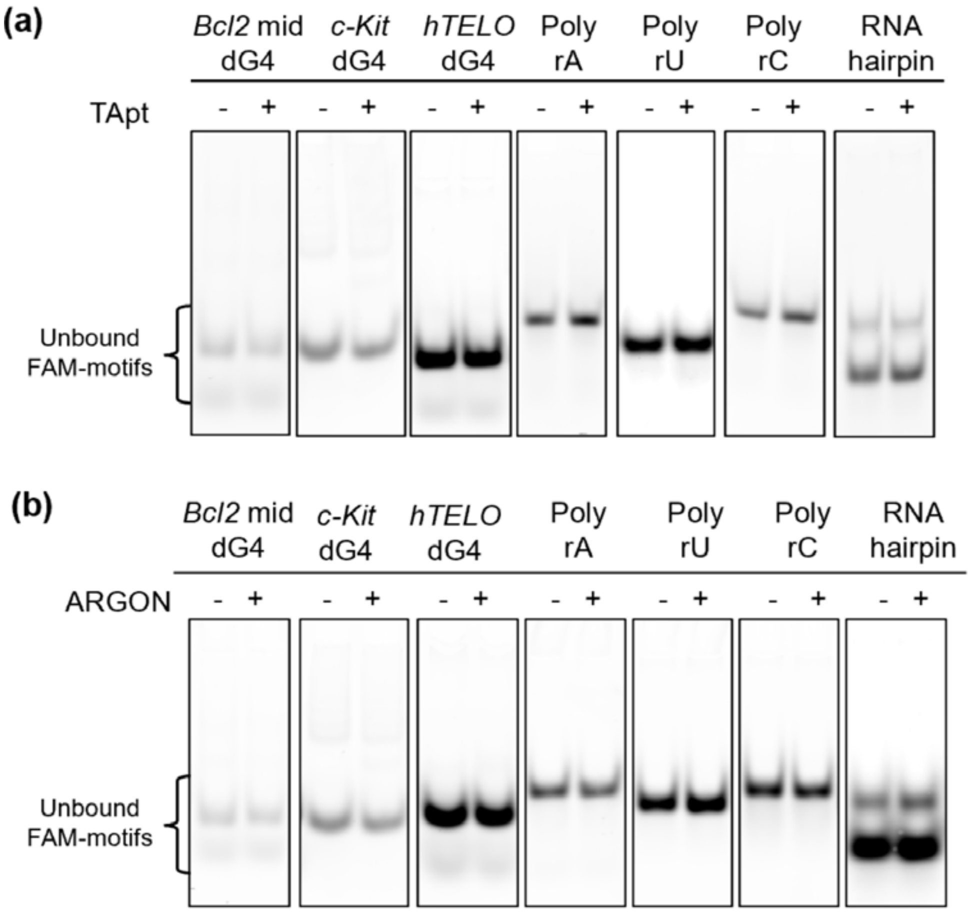

Figure S11. Binding selectivity of TApt and ARGON. 40 nM FAM-labeled non-target nucleic acid motifs were incubated with 400 nM (a) TApt or (b) ARGON in 10 mM GSH binding buffer. The lack of observable binding in these EMSA gels demonstrates the sequence and structural specificity of both TApt and ARGON for their intended rG4 targets.

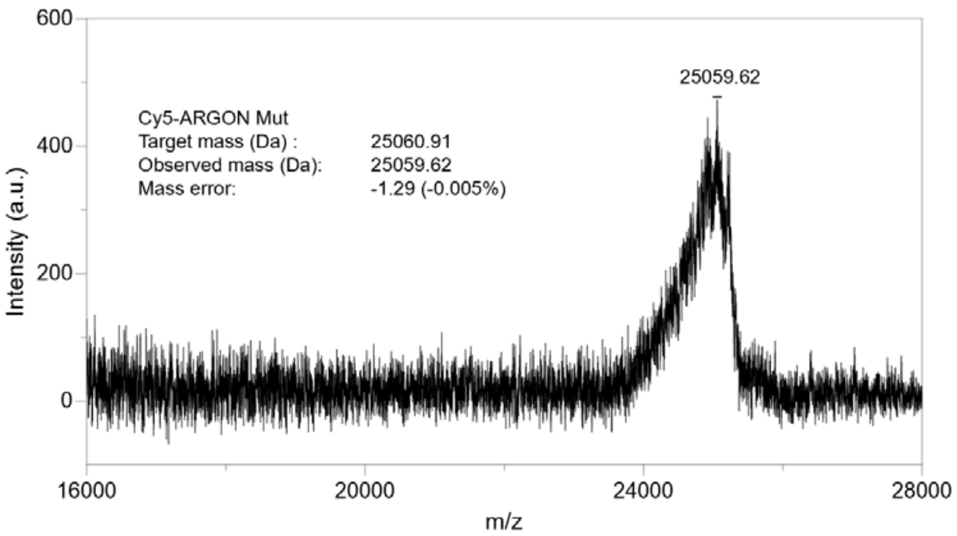

Figure S12. Mass spectra result of Cy5-ARGON Mut by MALDI-TOF assay. Mass calculated: 25060.91, mass found: 25059.62. This suggests that Cy5-ARGON Mut was synthesized successfully.

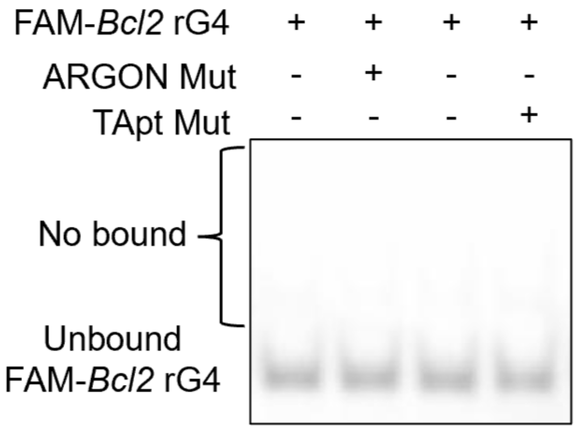

Figure S13. Binding specificity validation using mutant aptamers and wild-typed FAM-*Bcl2* rG4. FAM-*Bcl2* rG4 was incubated with ARGON Mut or TApt Mut in 10 mM GSH binding buffer. The lack of a gel shift demonstrates that mutations in the core G-tetrad of aptamer abolish binding, underscoring the specificity of the interaction.

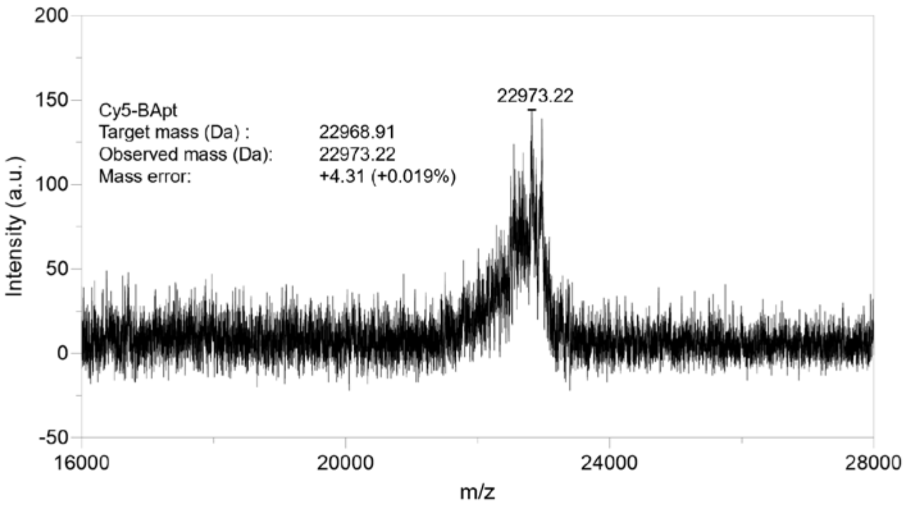

Figure S14. Mass spectra result of Cy5-BApt by MALDI-TOF assay. Mass calculated: 22968.91, mass found: 22973.22. This suggests that Cy5-BApt Mut was synthesized successfully.

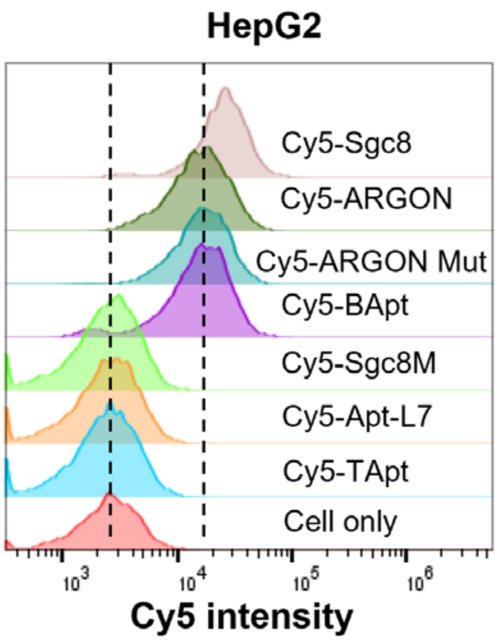

Figure S15. Internalization of molecules in HepG2 cells. Representative flow cytometry histograms showing the cellular uptake of Cy5-ARGON and control molecules in HepG2 cells after 4 h of incubation. The results are consistent with those in HeLa cells, confirming that the Sgc8-mediated internalization mechanism is generalizable to different PTK7-positive cell lines.

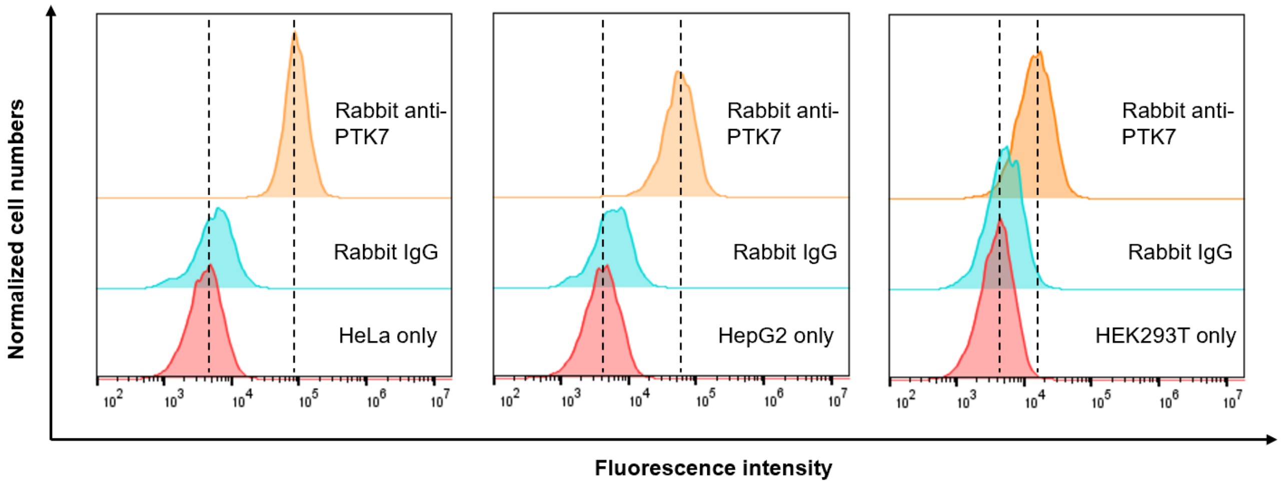

Figure S16. PTK7 receptor expression levels across cell lines. HeLa, HepG2, and HEK293T cells were stained with a rabbit anti-PTK7 antibody or an IgG control, followed by an Alexa Fluor 633-labeled secondary antibody, and analyzed by flow cytometry. The data confirm high PTK7 expression in HeLa and HepG2 cells, and low but detectable expression in HEK293T cells, providing the rationale for the observed differential internalization.

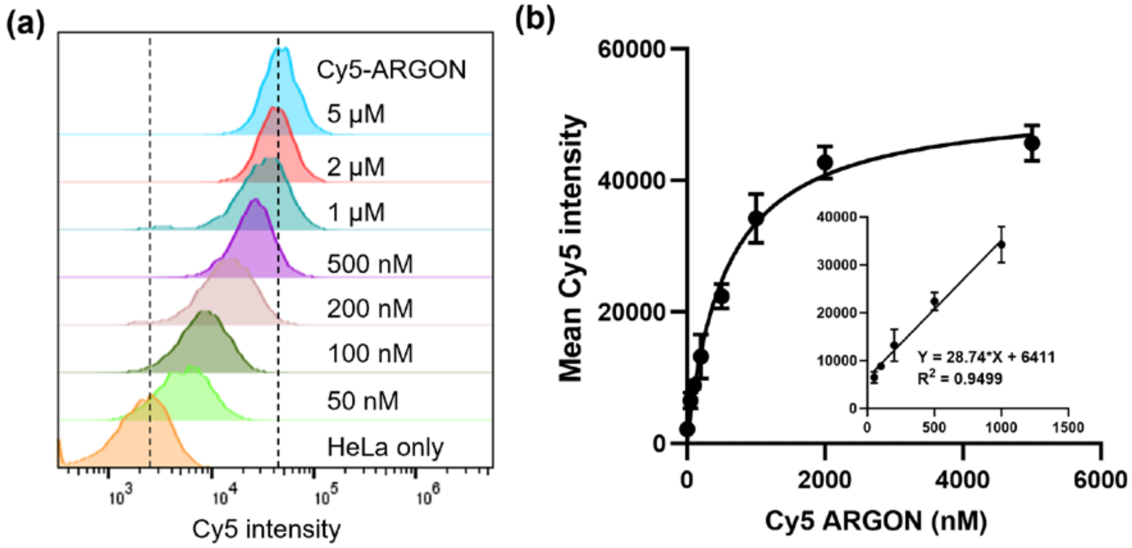

Figure S17. Dose-dependent cellular uptake and saturation kinetics of ARGON. (a) Representative flow cytometry histograms showing the intracellular fluorescence intensity of HeLa cells incubated with Cy5-labeled ARGON at concentrations ranging from 50 nM to 5 µM for 4 h. The shift in fluorescence intensity indicates progressive cellular internalization. **(b)** Quantification of mean Cy5 fluorescence intensity versus ARGON concentration. Data points represent the mean ± SD (n = 3). The uptake exhibits a linear correlation in the range of 50 nM to 1 µM, followed by a plateau at concentrations above 2 µM, indicating saturation of the cellular uptake machinery.

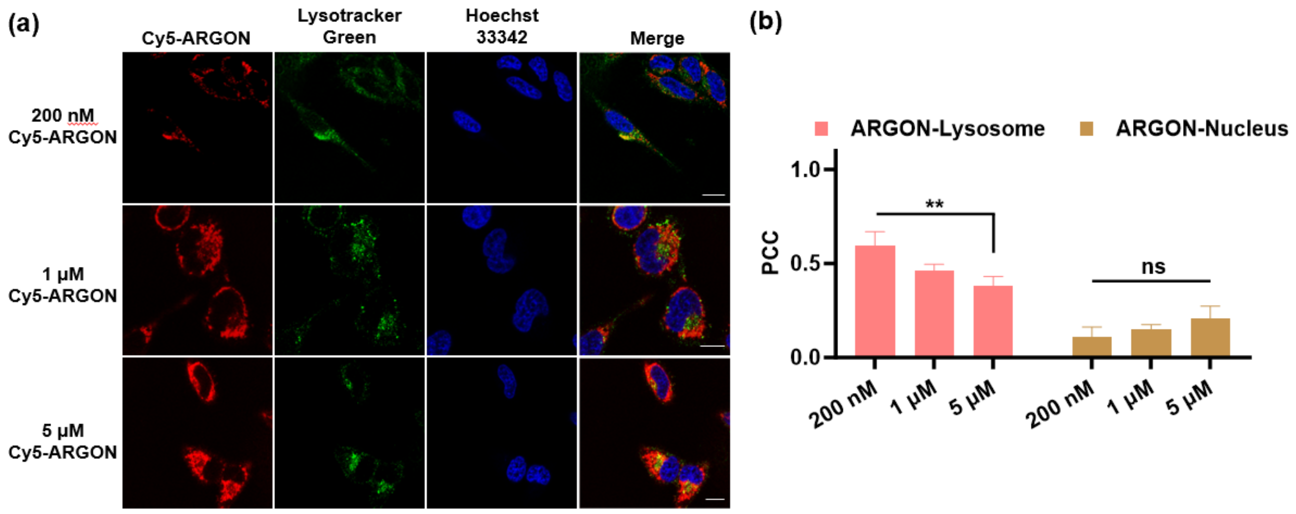

Figure S18. Subcellular localization and dose-dependent endosomal escape of ARGON. (a) Representative confocal microscopy images of HeLa cells treated with Cy5-ARGON at 200 nM, 1 μM, and 5 μM for 4 h. Cells were co-stained with LysoTracker Green to label lysosomes/endosomes and Hoechst 33342 to label nuclei. Scale bars: 10 μm. **(b)** Quantitative analysis of Pearson's Correlation Coefficient (PCC) between Cy5-ARGON and LysoTracker or Hoechst. All statistical data are mean ± SD (n = 3). Statistical significance: **P < 0.01, ns denotes no significant difference. At 200 nM, ARGON shows strong lysosomal co-localization. As the concentration increases to 1 μM and 5 μM, the PCC with lysosomes decreases, indicating partial overflow or escape from endo-lysosomal compartments into the cytoplasm. Conversely, PCC values with the nucleus remain negligible across all tested doses, confirming the absence of significant nuclear accumulation.

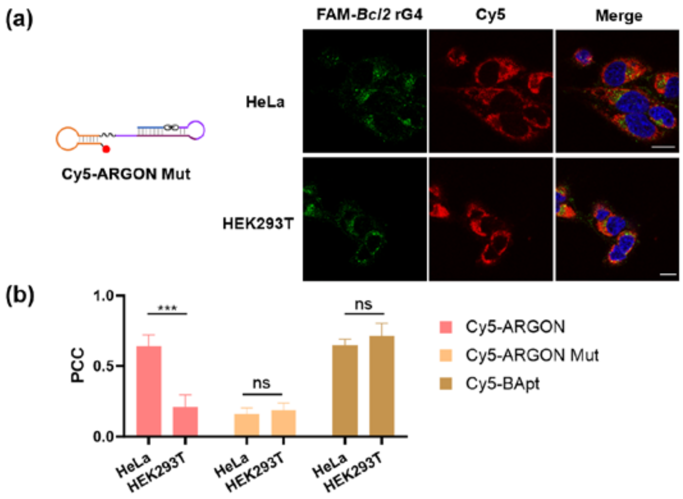

Figure S19. Cell confocal imaging post-treated with Cy5-ARGON Mut and colocalization analysis of three internalized molecules. (a) Confocal images of HeLa and HEK293T cells transfected with FAM-*Bcl2* rG4 (green) and incubated with Cy5-ARGON Mut (red). The final fields are merged with Hoechst 33342-stained nucleus. Scale bars = 10 μm. **(b)** Quantification of colocalization using Pearson's correlation coefficient (PCC). The low PCC values and lack of significant difference between cell lines confirm that the mutant aptamer does not specifically bind the target rG4, despite successful cellular entry. All data are mean ± SD (n = 3). Statistical significance: ***P < 0.001, ns denotes no significant difference.

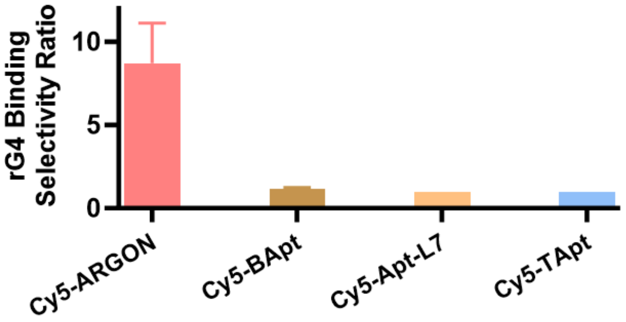

Input A (Sgc8)	1	1	0	0
Input B (Lock)	1	0	1	0
Output (distinguishable)	1	0	0	0

Figure S20. Quantitative analysis of rG4 binding selectivity between HeLa and HEK293T cells. rG4 binding selectivity ratio was calculated as % Colocalization (HeLa) / % Colocalization (HEK293T) based on Figure 3a. All statistical data are mean ± SD (n = 3). Only when dual modular inputs co-exist (Input 1,1), tumor cellular rG4 can be specifically targeted owing to distinguishable channel overlap and molecule binding between HeLa and HEK293T (Output 1).

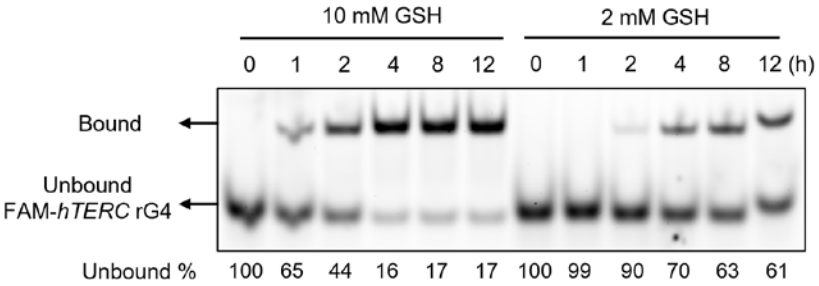

Figure S21. Binding kinetics of ARGON to FAM-*hTERC* rG4. EMSA analysis of 40 nM FAM-*hTERC* rG4 incubated with 400 nM ARGON in binding buffer containing 2 mM or 10 mM GSH. The faster and more complete complex formation in 10 mM GSH demonstrates the elevated GSH-responsive binding activation of ARGON towards the *hTERC* rG4 target.

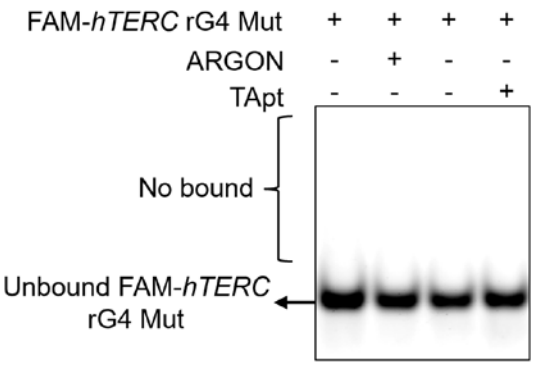

Figure S22. Binding specificity of ARGON and TApt for FAM-*hTERC* rG4. EMSA result was present after 40 nM FAM-*hTERC* rG4 Mut was incubated with 400 nM ARGON or TApt in 10 mM GSH binding buffer. The absence of a band shift confirms that binding to the *hTERC* rG4 is sequence-specific and requires an intact G-tetrad core in the target.

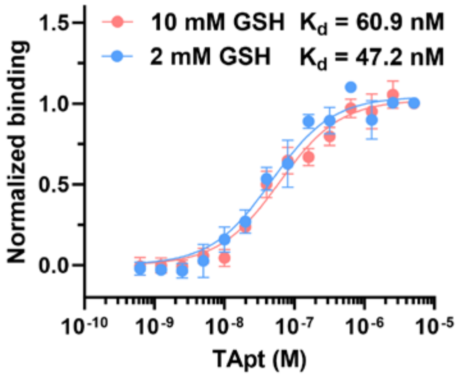

Figure S23. MST binding analysis of TApt with FAM-hTERC rG4. MST-derived binding curves for FAM-*hTERC* rG4 titrated with TApt in binding buffer containing 2 mM or 10 mM GSH. All statistical data are presented as mean ± SD (n = 3). The similar high affinities observed under both conditions confirm that the binding of the parent aptamer (TApt) is constitutive and not dependent on GSH concentration.

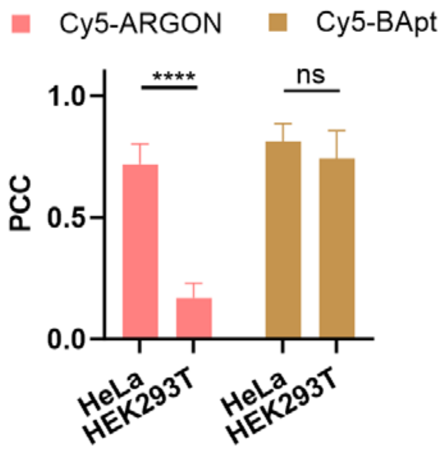

Figure S24. PCC analysis of the confocal images of Figure 4d. PCC quantified the overlap situations between the Cy5 (ARGON or BApt) and FAM (*hTERC* rG4) channels in HeLa and HEK293T cells. All data are mean ± SD, n = 3. Statistical significance: ****P < 0.0001, ns denotes no significant difference. The highly distinguishable PCC value specifically for ARGON in HeLa cells validates the AND-gated activation mechanism for binding *hTERC* rG4.

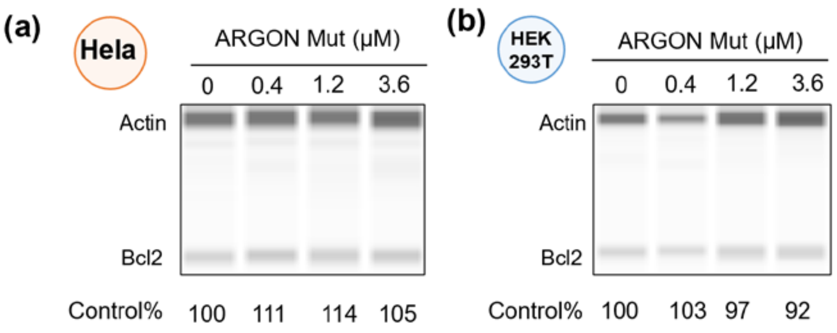

Figure S25. Bcl2 protein levels after treatment with ARGON Mut. Capillary electrophoresis-based Simple Western™ analysis of **(a)** HeLa and **(b)** HEK293T cells treated with different concentrations of the non-binding control, ARGON Mut, for 48 h. The minimal change in Bcl2 protein levels confirms that the functional effects of wild-type ARGON are due to specific rG4 binding and not nonspecific toxicity.

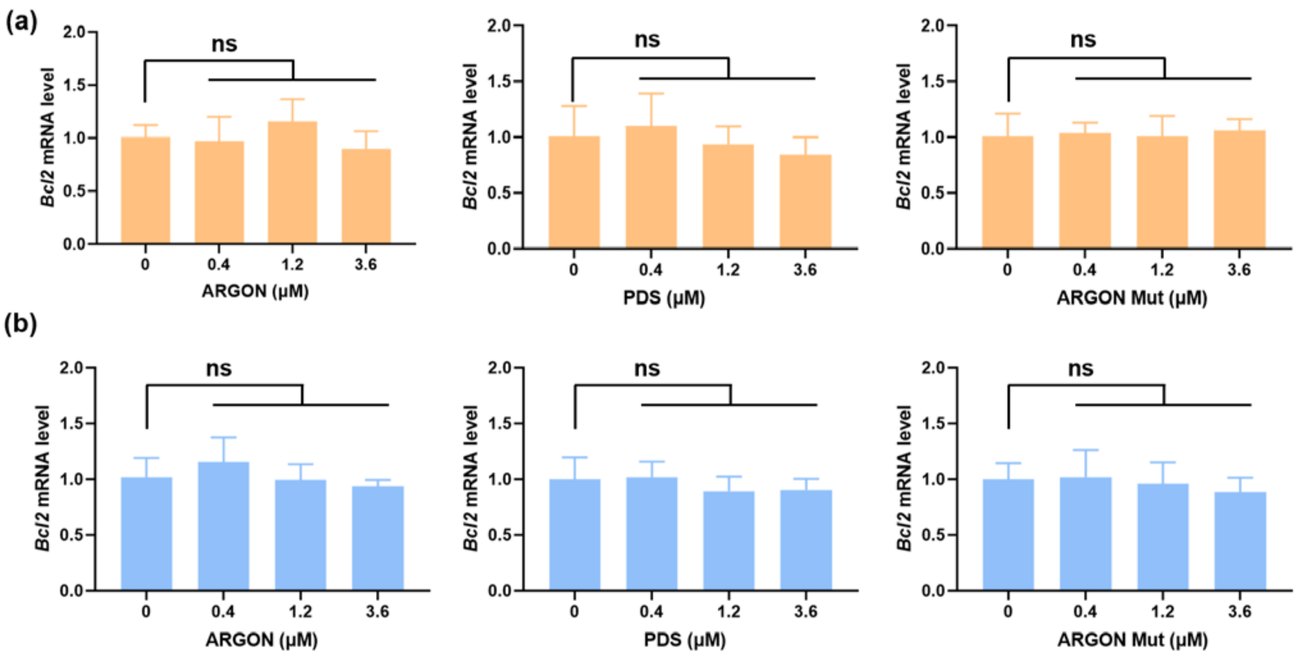

Figure S26. *Bcl2* mRNA levels post-treatment with ARGON and control molecules. RT-qPCR analysis of *Bcl2* mRNA in **(a)** HeLa and **(b)** HEK293T cells after 48 h incubation with different molecules. All statistical data are presented as mean ± SD (n = 3). Statistical significance: ns denotes no significant difference. The lack of significant change indicates that ARGON and other treatments regulate Bcl2 expression at the translational level, consistent with the mechanism of rG4 stabilization in the 5' UTR.

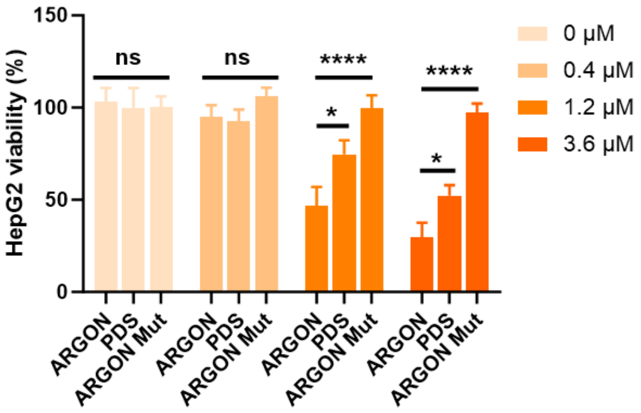

Figure S27. HepG2 cellular viability after treatment with different concentrations of molecules for 72 h. All statistical data are mean ± s.d., n = 3. Statistical significance: ****P < 0.0001, *P < 0.05, ns denotes no significant difference. The results showed ARGON has higher inhibition rate to HepG2 cell proliferation than positive control PDS and that negative control, ARGON Mut did not show any regulation effect to HepG2 cell proliferation. These results indicated ARGON performed high inhibition efficiency and specificity to the cell proliferation of target HepG2 cells.

Uncropped gels and blots for main and supporting figures

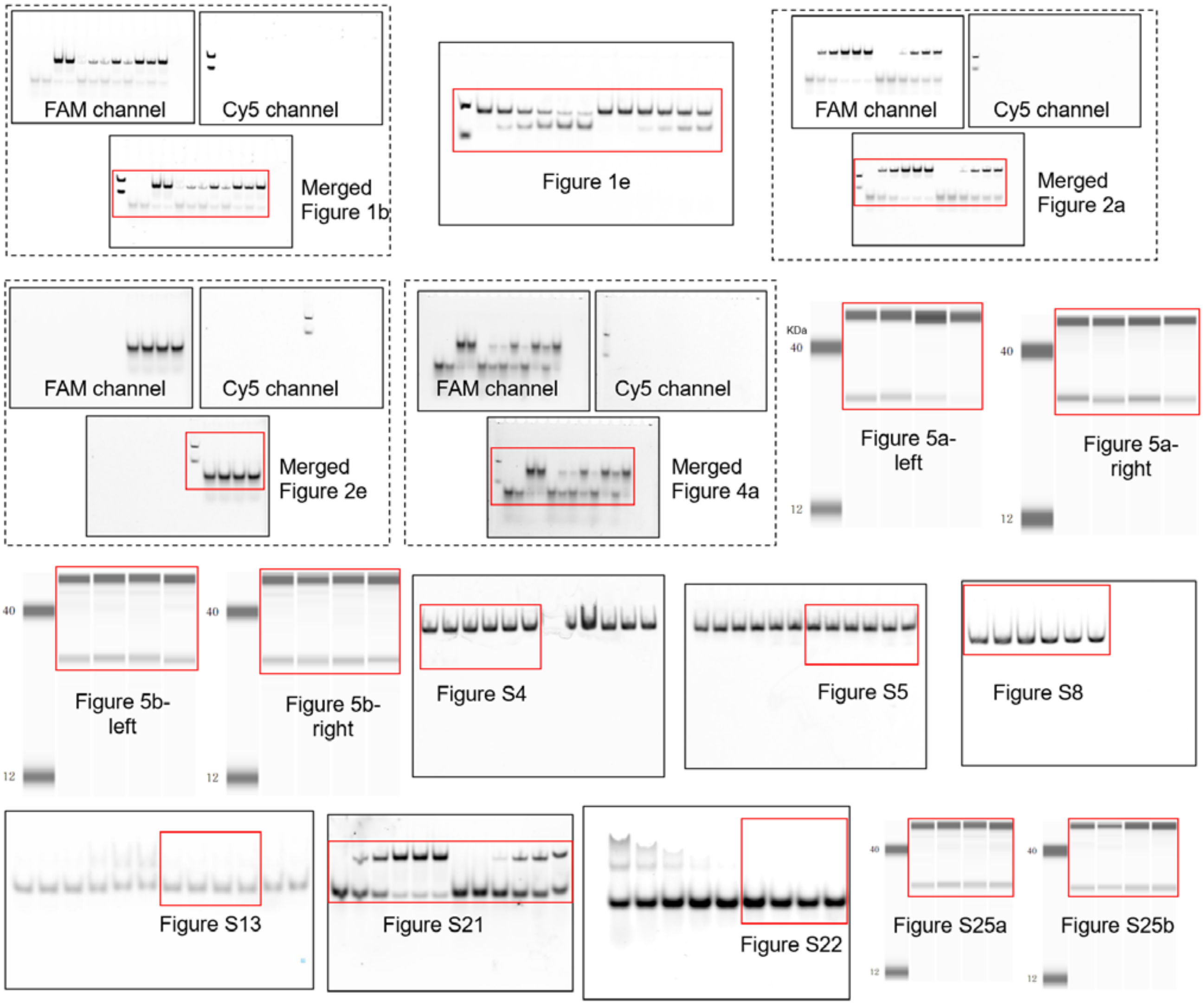

3. Supporting Tables

Table S1. DNA and RNA sequences used in this work.

Name	Sequence (5'-3')	Modification or note	Type
Bcl2 rG4	FAM-GGGGGCCGUGGGGUGGGAGCUGGGG	5’ FAM	D-RNA
L-Apt.4-1c (TApt)	GCCCUAAAGGUGGUGGUGGGAGGGC	5’ N_3 or 5’ Cy5	L-RNA
Apt-L11	N_3-GCCCUAAAGGUGGUGGUGGGAGGGC-S-S-CACCACCACCU	5’ N_3, disulfide bond linker	L-RNA
Apt-L9	N_3-GCCCUAAAGGUGGUGGUGGGAGGGC-S-S-CACCACCAC	5’ N_3, disulfide bond linker	L-RNA
Apt-L7	GCCCUAAAGGUGGUGGUGGGAGGGC-S-S-CACCACC	5’ N_3, disulfide bond linker; or 5’ Cy5, disulfide bond linker	L-RNA
Apt-L5	N_3-GCCCUAAAGGUGGUGGUGGGAGGGC-S-S-CACCA	5’ N_3, disulfide bond linker	L-RNA
Sgc8	ATCTAACTGCTGCGCCGCCGGGAAAATACTGTACGGTTAGA-DBCO	5’ Cy5, 3’DBCO; or 3’DBCO	D-DNA
ARGON	ATCTAACTGCTGCGCCGCCGGGAAAATACTGTACGGTTAGA-DBCO-N_3-GCCCUAAAGGUGGUGGUGGGAGGGC-S-S-CACCACC	5’ Cy5, or fluorescence group-free; synthesized from Apt-L7 and Cy5-Sgc8	D-DNA + L-RNA

Bcl2 rG4 Mut	FAM-GAGAGCCGUGAAGUAAGAGCUGAAG	5’ FAM	D-RNA
hTERC rG4 Mut	FAM-GAAUUGCGGAGAAUGAACCU	5’ FAM	D-RNA
Bcl2 mid dG4	FAM-GGGCGCGGGAGGAAGGGGGCGGG	5’ FAM	D-DNA
c-Kit dG4	FAM-AGGGAGGGCGCTGGGAGGAGGG	5’ FAM	D-DNA
hTELO dG4	FAM-TTAGGGTTAGGGTTAGGGTTAGGG	5’ FAM	D-DNA
Poly rA	FAM-AAAAAAAAAAAAAAAAAA	5’ FAM	D-RNA
Poly rU	FAM-UUUUUUUUUUUUUUUUUU	5’ FAM	D-RNA
Poly rG	FAM-CCCCCCCCCCCCCCCCCC	5’ FAM	D-RNA
RNA hairpin	FAM-CAGUACAGAUCUGUACUG	5’ FAM	D-RNA
Apt-L7 Mut	N_3-GCCCUAAAGAUGAUGAUGAGAGGGU-S-S-CAUCAUC	5’ N_3, disulfide bond linker	L-RNA
ARGON Mut	Cy5-ATCTAACTGCTGCGCCGCCGGGAAAATACTGTACGGTTAGA-DBCO-N_3-GCCCUAAAGAUGAUGAUGAGAGGGU-S-S-CAUCAUC	Cy5 label; synthesized from Apt-L7 Mut and Cy5-Sgc8	D-DNA + L-RNA
BApt	Cy5-ATCTAACTGCTGCGCCGCCGGGAAAATACTGTACGGTTAGA-DBCO-N_3-	Cy5 label; synthesized from L-Apt.4-1c and Cy5-Sgc8	D-DNA + L-RNA

	GCCCUAAAGGUGGUGGUGGGAG GGC		
Sgc8M	Cy5-ATCTAACTGTTTTTTTTTTTTTTTTT TTTTTTTCGGTTAGA	5’ Cy5	D-DNA
hTERC rG4	FAM-AAAGGGUUGCGGAGGGUGGGCC U	5’ FAM	D-RNA
Bcl2-FP	ATCGCCCTGTGGATGACTGAGT	Forward primer for RT-qPCR	D-DNA
Bcl2-RP	GCCAGGAGAAATCAAACAGAGGC	Reverse primer for RT-qPCR	D-DNA
GAPDH-FP	GGAGCGAGATCCCTCCAAAAT	Forward primer for RT-qPCR	D-DNA
GAPDH-RP	GGCTGTTGTCATACTTCTCATGG	Reverse primer for RT-qPCR	D-DNA

Table S2. Physicochemical properties of the ARGON and main ARGON controls in this study.

Name	Nucleotide length	Molecular weight (Da)	Composition	Cost (USD / 5 OD)
Cy5-ARGON	73	25124.91	Cy5-Sgc8-DBCO, N_3-Apt-L7	300
ARGON	73	24466.11	Sgc8-DBCO, N_3-Apt-L7 Mut	270
Cy5-ARGON Mut	73	25060.91	Cy5-Sgc8-DBCO, N_3-Apt-L7 Mut	300
Cy5-BApt	67	22968.91	Cy5-Sgc8-DBCO, N_3-TApt	230

Table footnote: 1) Disulfide bonds were introduced via commercially standard disulfide phosphoramidites during automated solid-phase synthesis, requiring no additional post-synthetic liquid-phase steps. 2) All molecules above were obtained by commercially available solid-phase synthesis and then click reaction. 3) The estimated research-scale cost for the fully functionalized Cy5-ARGON is only ~$300 per 5 OD. This is highly economical for a multifunctional probe containing L and D-nucleotides, disulfide linkage, fluorophores, and click handles, making it accessible for widespread research use.

Table S3. Truth table of AND logic gate by studying the FAM-*Bcl2* rG4 colocalization.

**Input A**	**Input B**	**rG4 Binding Selectivity Ratio**	**Output**	**Logic state**	**Result**
1	1	8.6	1	ON	Highly distinguishable binding
1	0	1.1	0	OFF (maximal leakage)	Indistinguishable binding
0	1	1.0	0	OFF	No binding
0	0	1.0	0	OFF	No binding

Table footnote: 1 and 0 in Input denote presence and absence respectively. rG4 Binding Selectivity Ratio = %Colocalization in Target Cells (HeLa) / %Colocalization in Non-Target Cells (HEK293T). When the Ratio is nearly 1, the logic output is 0 (OFF state); while the Ratio is much greater than 1, the logic output is 1 (ON state). For inputs (0,0) and (0,1), the molecules can hardly enter the cells due to the lack of Sgc8, resulting in negligible colocalization in both cell lines and a ratio of ~1.

4. References

[1] I. Esain-Garcia, A. Kirchner, L. Melidis, *et al.*, "G-quadruplex DNA structure is a positive regulator of MYC transcription," *Proc. Natl. Acad. Sci. U. S. A.* **2024**, 121, e2320240121.

[2] X. Zhang, J. Spiegel, S. Martínez Cuesta, S. Adhikari, S. Balasubramanian, "Chemical profiling of DNA G-quadruplex-interacting proteins in live cells," *Nat. Chem.* **2021**, 13, 626-633.

[3] M. Zuker, "Mfold web server for nucleic acid folding and hybridization prediction," *Nucleic Acids Res.* **2003**, 31, 3406-3415.

[4] N. R. Markham, M. Zuker, "DINAMelt web server for nucleic acid melting prediction," *Nucleic Acids Res.* **2005**, 33, W577-W581.
